# Science maps for exploration, navigation, and reflection—A graphic approach to strategic thinking

**DOI:** 10.1371/journal.pone.0262081

**Published:** 2021-12-31

**Authors:** Flemming Skov

**Affiliations:** Department of Ecoscience, Aarhus University, Aarhus, Denmark; Universitat de Barcelona, SPAIN

## Abstract

The world of science is growing at an unprecedented speed with more and more scholarly papers produced each year. The scientific landscape is constantly changing as research specialties evolve, merge or become obsolete. It is difficult for researchers, research managers and the public alike to keep abreast with these changes and maintain a true and fair overview of the world of science. Such an overview is necessary to stimulate scientific progress, to maintain flexible and responsive research organizations, and to secure collaboration and knowledge exchange between different research specialties and the wider community. Although science mapping is applied to a wide range of scientific areas, examples of their practical use are sparse. This paper demonstrates how to use a topical, scientific reference maps to understand and navigate in dynamic research landscapes and how to utilize science maps to facilitate strategic thinking. In this study, the research domain of biology at Aarhus University serves as an example. All scientific papers authored by the current, permanent staff were extracted (6,830 in total). These papers were used to create a semantic cognitive map of the research field using a co-word analysis based on keywords and keyword phrases. A workflow was written in Python for easy and fast retrieval of information for topic maps (including tokens from keywords section and title) to generate intelligible research maps, and to visualize the distribution of topics (keywords), papers, journal categories, individual researchers and research groups on any scale. The resulting projections revealed new insights into the structure of the research community and made it possible to compare researchers or research groups to describe differences and similarities, to find scientific overlaps or gaps, and to understand how they relate and connect. Science mapping can be used for intended (top-down) as well as emergent (bottom-up) strategy development. The paper concludes that science maps provide alternative views of the intricate structures of science to supplement traditional bibliometric information. These insights may help strengthen strategic thinking and boost creativity and thus contribute to the progress of science.

## Introduction

Science evolves and changes rapidly over time. The annual production of scientific articles grows almost exponentially and doubles every 9–15 years [1, 2]. New research specialties and topics emerge, existing topics fuse or become obsolete and boundaries and compartments within the research landscapes change constantly [3, 4]. As a result, it becomes increasingly difficult to maintain a good overview of even relatively small research fields. In the light of pressing global problems, the scientific community needs tools to stimulate research, to prioritize the development of research fields, to recruit staff and reviewers and to evaluate the impact of science on society [5].

### Keeping up with change–strategic planning or strategic thinking?

Universities are not static organizations; they must constantly adapt to the world and compete for private or governmental funding, for staff and students, and try to live up to public expectations of excellent higher education, scientific innovation, new discoveries and solutions to pressing societal problems. A constantly shifting scientific landscape is a special challenge for research institutions since the best organization of staff today may be suboptimal and out-of-date tomorrow. Most universities have cycles of strategic planning where long- and short-term goals are set, where plans and strategies are formulated, and where the organizational structure is evaluated and perhaps updated. There are many approaches to strategy development and it’s outside the scope of this paper to describe them in detail, but two schools of thought are generally recognized [6].

Strategic planning (mainly associated with Michael Porter [7]) is a strict, analytical approach based on strategic programming. Strategic planning is commonly initiated top down as a formalized process with clear rules and protocols based on analysis of performance metrics and benchmarking. Strategic planning is usually grounded in the existing organization and aims at setting measurable and realistic goals and to define detailed plans and workflows to reach those goals.

Strategic thinking as promoted by Henry Mintzberg [8], focusses on the creative and synthetic aspects of strategy development. Mintzberg argues that much more emphasis should be put on strategic thinking in the initial phases of strategy development. Some form of planning is obviously necessary, but strategy development, in his view, has to be an open-ended and engaging process that involves all sorts of knowledge and input and not just ‘hard’ facts and numbers. Strategic thinking requires intuition and creativity and helps stimulate the development of new ideas and solutions [9]. Mintzberg sees strategy as an ever-ongoing process and not just the usual step-by-step plans for the next strategy period. In its most extreme form this type of strategy development is emergent (ad-hoc) rather than intended as strategic planning is. Therefore, there are no exact procedures for strategic thinking, but various approaches are described in the literature [10, 11].

In a discussion of strategy processes, Heracleous [6] proposes a dialectical view of strategic thinking and strategic planning where both approaches are valuable and complement each other. He uses an analogy for learning and finds that strategic planning corresponds to ‘single-loop’ learning, where errors or unexpected results are dealt with in the existing framework and work modes. Strategic thinking, on the other hand, is closer to the concept of ‘double-loop’ learning [12] as it also challenges assumptions and norms and thus the current organizational structure and its workflows and goals.

The procedures and data requirements for traditional strategy planning are well known and this study will focus on the potential use of science mapping to generate new views and insights to stimulate strategic thinking.

### How science maps may improve strategic thinking

A solid understanding of the literature is necessary to provide a comprehensive view of a scientific field. The study of bibliographic data is known as bibliometrics and the field has two main approaches: performance analysis of productivity (e.g., number of papers, number of citations, journal impact, and h-index) and science mapping [13, 14]. Performance analyses based on bibliometric data are regularly used to govern and manage science at higher administrative levels where allocation of resources often depends on various performance indicators (e.g., university rankings or number of publications in journals with high impact factor) and where recruitments and promotions are based on the h-index or similar indicators. It is problematic, however, that the evaluation of research today is controlled primarily by data and not by judgement. In their ‘*Leiden Manifesto for research metrics’* [15], Hicks et al. discuss some of the problems that arise, when evaluation is based excessively on simple quantitative indicators. The manifesto states that quantitative evidence as well as qualitative judgement are necessary in decision-making concerning science management. For example, in order to measure performance indicators against research missions or societal needs, or to protect excellence in locally relevant research.

As the number of articles within a given field grows methods are needed to deliver a macro view of what is going on scientifically, to map patterns of activity, and to understand in what direction science is moving. Science mapping is a useful approach for this purpose and there are many excellent examples in the literature [16–25]. The use of science maps may improve understanding by visualizing the structure and content of a scientific research specialty. Science maps show how the units of research (keywords, papers, researchers, and research groups) are organized, linked and interconnected [4, 22]. Maps of science provide a bird’s eye view of the scientific landscape and have been useful tools for understanding the structure of science and the spread and interconnection of disciplines [26]. A wide variety of methods and approaches have been used to construct science maps. The VOS viewer (http://www.vosviewer.com) [27] is a common tool and has been used to map many different domains such as biodiversity [28], the environmental footprint [29], demolition waste management [30], computer graphics [13], entrepreneurship [31], Alzheimer’s disease [32], depression related diseases [33], natural disaster research [34], climate change [35], Mediterranean studies [36] and finally the topic of science mapping itself [37]. A more recent tool–‘*Bibliometrix’*–is available as an R-script for science mapping [38] and the code can be downloaded and modified to fit specific needs. Science mapping uses a variety of other approaches including network analysis based on collaboration [39–44] or topics that focus on the structure of scientific ideas [45, 46]. For a thorough review see [37] or [19]. Science maps are visually attractive and generally appreciated, but are rarely used for practical purposes. The pure science map (keywords in a 2D co-term universe) is, despite its apparent simplicity and the overview it provides, often quite difficult to read, understand and navigate in. The use of proper overlay techniques where ‘landmarks’ such as researchers, research groups, sciences subject categories are mapped upon the reference map may significantly improve the understanding of the map and in this manner support its use for strategic thinking [47]. This paper explores how science mapping combined with visual presentation of facts and performance metrics may improve strategic thinking and thus strategy development. Available ‘hard’ data commonly used to describe scientific organizations (e.g., staff composition and productivity: age, gender, positions, number of papers, h-index, etc.) are extracted and analyzed. This information is then presented visually in the context of a science map that shows how researchers and research groups are related and interconnected.

The scientific research domain of biology at Aarhus University has recently gone through considerable organizational changes of merging and splitting and is an interesting test case. Today, biological research at Aarhus University resides mainly within two departments, Dept. of Biology and Dept. of Ecoscience, respectively (hereafter referred to as ‘*BIOL’* and ‘*ECOS’*) Biology related research exists elsewhere at the university (e.g., Department of Environmental Science, Department of Biochemistry, and the three departments doing agro-related research), but only to a minor degree and these departments are not included in the present study.

The aim of this study is to demonstrate how the construction and use of a science map may provide a sense of overview and outline and visualize the distribution of entities and their relationships. Specifically, the paper explores how science mapping may stimulate associative and creative thinking as an input to strategic planning. This is feasible through exploration (examining the distribution and relationship of scientific entities such as keywords, papers, researchers or groups in the landscape); navigation (practical use of the tool to visualize competences for, e.g., teaching and societal needs); and finally for reflection and creative thinking as a help to discover new patterns, generate ideas and innovative solutions. Finally the paper proposes to use science maps as a sort of board game or playing surface on which to develop, demonstrate and debate various options and scenarios for strategic thinking.

## Methods

Information about the permanent staff of BIOL and ECOS was extracted in June 2020. Data included only basic and non-sensitive information such as name, age, gender, job title, affiliation (section and department) and Researcher-ID if available. Names were only used to extract bibliometric data if a Researcher-ID was missing, but not shown in text or figures. The map making and domain visualization used in this study followed the basic steps described in (4): (I) evaluation of data sources and subsequent data extraction; (II) selection of units of analysis and of measures; (III) data layout (calculation of similarity between units and ordination); and (IV) mapping and visualization.

### Data extraction

Bibliometric data records were imported from Clarivate Web of Science (https://www.webofscience.com/) as txt-files. The raw material (corpus) for the analysis was the total number of scientific papers published by the current permanent scientific staff. Records were retrieved by Researcher-ID when possible. To find publications from staff without a Researcher-ID (or where it had not been updated), a more basic search strategy was applied using name and affiliation. This is potentially more error prone as author names (and even combinations of names and affiliation) may be identical and this may lead to the inclusion of papers to the corpus that do not belong there. In some cases, therefore, it may be difficult to create a full list of publications, but experience showed that the final maps were not particularly sensitive to imperfect sampling when the analysis was based on a sufficiently large number of scientific papers (own observation and see also [2]).

### Units of analysis

Presently, scholarly knowledge exists primarily as written documents of which the scientific paper is the most important. The analyses in this study were based on a collection of papers and used the fundamental units suggested by [4, 22, 25, 48]. Papers appear in journals that often cover a certain research specialty. Papers in the same journal are thus (to some degree at least) related by scope. Most journals use keywords to summarize the scientific content of a paper. Keywords are often directly accessible as author keywords, but can also be extracted from paper titles or abstracts using text mining techniques. The units used in this study were papers, the journal they were published in, their authors and the keyword terms used to describe them. A paper also contain citations or references that represents links to existing knowledge, but an analysis of the network of citations was not included in this study.

### Data layout

The main purpose of this study was to create a semantic or cognitive map of the field of biology at Aarhus University using a co-word analysis (also known as a term-based map [25]). The input to the analysis were keywords or rather keyword phrases as they often consist of one or more words. Keyword phrases are more precise than single keywords and contain more information [2]. The keyword phrase ‘*temperature-stress*’, e.g., is more specialized and thus more informative than the two keywords used separately (‘*temperature’* and ‘*stress’*). In the following, the term keyword is used for single as well as multiple keyword phrases. The first step of the approach was to build a co-term occurrence matrix linking keywords that appear together in the same paper (either as author-supplied keywords, Web of Science Keyword Plus, or in the title of the paper). Before constructing the co-occurrence matrix, keywords (including individual parts of keyword phrases) were cleansed using Porter’s suffix stripping [49]. The resulting matrix is a n-dimensional co-occurrence network where n is the number of unique keywords in the corpus. In such a matrix, each keyword is a node and all co-occurrences of two keywords are links. The weight or strength of the link is proportional to the number of times the two keywords occur together. If a measure of co-occurrence varies between the units of the analysis, it is common practice to normalize data to transform them to a common scale [25]. An initial evaluation of the raw data used for this study, however, did not show any distinct trends or biases in number of keywords per article between years or among Web of Science subject categories. Since the aim of the study was to create a general map for one unit only, the number of co-occurrences between two keywords served as a measure of attraction (weight) between them without further scaling.

### Mapping and visualization

**Fig 1:**
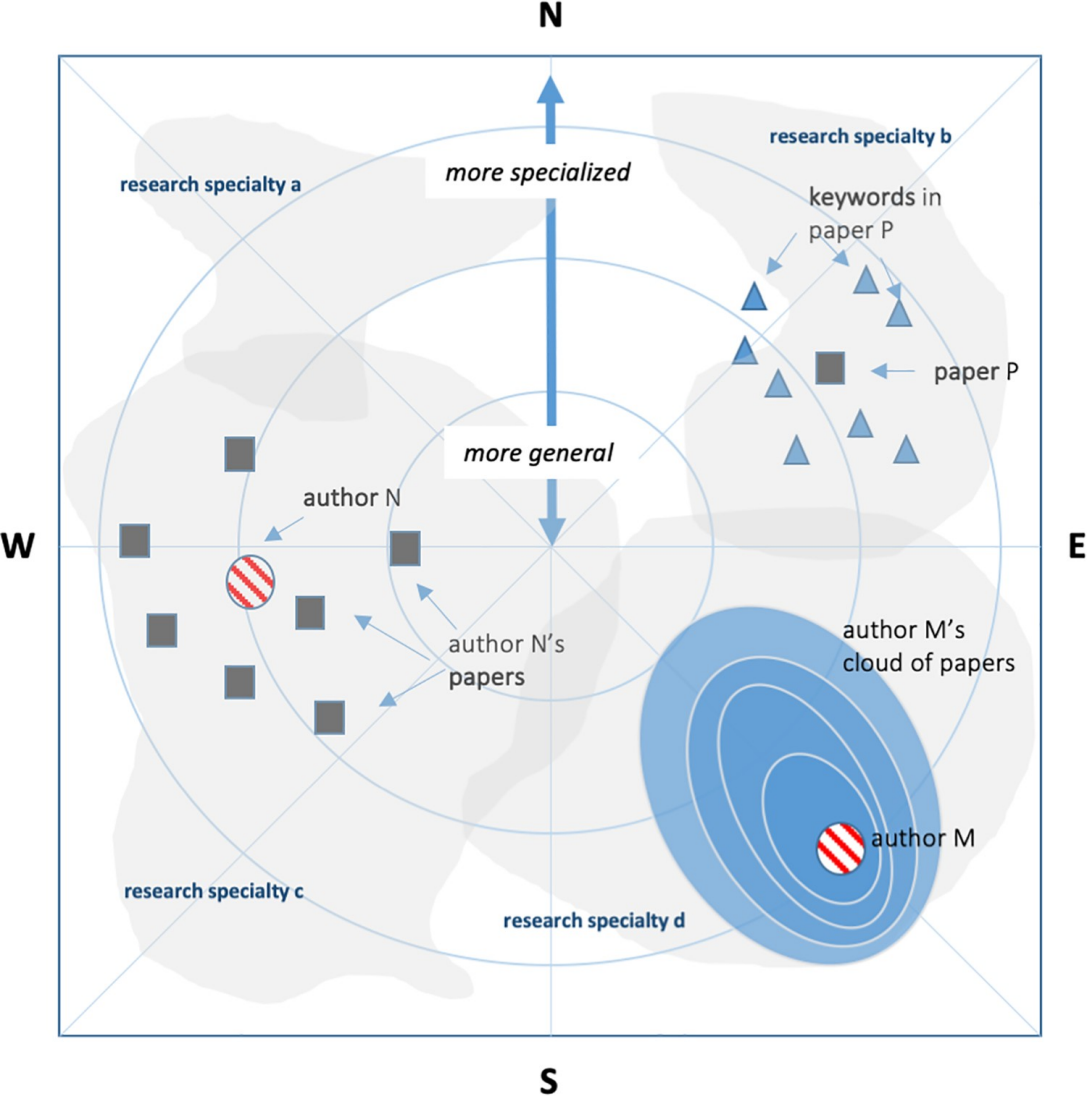
Main features of the science map. In this example, keywords are shown as triangles, papers as squares, authors as circles and research specialties as diffuse clouds. Each keyword has a fixed position in the reference map. The most general keywords that are shared by many research specialties are found near the center of the map. The further towards the periphery, the more characteristic a keyword is. Keywords may be clustered into fuzzy research specialties (denoted a, b, d, and d here). The center of a paper is placed in the center of the keywords it contains. The center of an author is located in the center of his or her papers. researcher (or a research group) is better represented by a continuous field that maps the density of articles. As a familiar analogy to an ordinary geographic map, directions may be denoted north, south, east, or west to help pinpoint certain locations in the map. Paper P in the map, for example, is located towards the north-east at a medium distance from the center. Author M is situated towards the south-east with a relatively narrow distribution of papers.

There are many techniques available to convert a high-dimensional dataset to a useful representation in just two dimensions (i.e., a map). Among the most common techniques are factor analysis and principal components analysis, multidimensional scaling, latent semantic analysis, pathfinder network scaling, and artificial neural network techniques such as self-organizing maps [4, 25]. In this study, a force directed placement (FDP) was used to obtain a suitable spatial configuration. A FDP has a physical analogy, where nodes are seen as bodies and edges as springs that provide attractive forces between them. Nodes are allowed to move according to the forces until a local energy minimum is realized [50]. In the FDP process, each keyword receives a set of 2D coordinates in the cognitive space. Keywords that often appear in the same papers will be close in the cognitive space as well. Keywords with a high degree of betweenness and centrality (i.e., keywords used commonly such as *impact*, *response*, *pattern*, *dynamics*, *distribution*, or *model*) tend to be found in the center of the map while rarer and more specific keywords will be located closer to the periphery. The latter are more characteristic of specific sub-disciplines or research specialties. The coordinates obtained in the FDP were used to map the position of keywords directly, but all other units (papers, researchers, groups of researchers, journals) were projected on the map using scientometric overlay mapping [51]. Overlays basically come in two forms [52]: as discrete objects or as continuous fields. When mapped as a discrete object, a symbol represents a unit in the center of its distribution. The discrete position of an article, for example, was calculated as the mean x-y coordinates of all keywords it contained and will thus be found in the center of this ‘cloud’ of keywords. This is clearly an oversimplification and does not represent the full cognitive space it fills, but is still useful in simple maps for overview. Correspondingly, the center location of a researcher was defined as the center of his or her articles. The position of a group of researchers and even the distribution of a subject area was calculated in the same way. The use of continuous fields makes it possible to visualize the shape of the cognitive space filled by the unit and provide more insight and information. Continuous fields were in this study implemented with a simple kernel density estimator. A continuous field or a profile map for an author, for example, shows the density of articles written by the author in the cognitive space and gives the viewer a much more accurate picture of which research specialties he or she cover. See Fig 1 for an explanation of the features discussed above.

All individual steps in the process from data cleaning, extraction of data, data layout and map visualization were programmed in Python and the scripts are available at Gitlab (https://gitlab.com/flemmingskov/plosone_science_map). The scripts are compatible with Phyton vs. 3.6 running in the data-science toolkit Anaconda (http://www.anaconda.com/https://anaconda.com). In addition to the standard setup, the following libraries are used: pandas (general data handling), sqlite3 for data base management, igraph (for network analysis), matplotlib and seaborn for data visualization, and nlkt (natural language toolkit for python (https://nlkt.org). Scripts are combined in a user-friendly front-end app using Streamlit (https://www.streamlit.io/)

A collection of articles authored by the current permanent staff at the two departments was the only input to the analysis. The full process involved the following steps each implemented as a python script in order to:

1. import text files from Web of Science and do basic data cleansing
2. create a vocabulary of unique keywords from author keywords and keyword plus
3. find and extract keywords from title and abstract using the vocabulary created in step 2
4. build a co-occurrence matrix for keyword
5. reduce dimensionality using FDP (force directed placement)
6. adjust and fine-tune the reference map for better presentation
7. prepare data for scientometric overlay mapping (here papers and authors are assigned coordinates in the map space), and finally
8. a mapping module to create actual maps and images.

## Results

### A brief history of biology at Aarhus University

The first version of BIOL was founded in 1992 at Aarhus University as a merger of five smaller biological departments. ECOS was originally part of the National Environmental Research Institute (NERI) affiliated to the Danish Ministry of the Environment and Energy and did independent strategic and applied research. NERI’s mission was to provide the scientific basis and environmental information for political and administrative decisions regarding nature and the external environment. On January 1^st^ 2007, NERI became part of Aarhus University as an academic area (more or less similar to a faculty). This was a result of a government decision to integrate sectorial- and university research to create fewer but larger research institutions in Denmark and thus obtain critical mass. In March 2011, Aarhus University went through a major reconstruction where rector reduced the number of main academic areas from nine to four and the number of departments from 55 to 26. This process resulted in a large Department of Bioscience as a merger of the Biology Department and biology research groups from the former National Environmental Research Institute (NERI) under the Faculty of Science and Technology. In 2019 the rectorate decided to split the very large Faculty of Science and Technology in two faculties (Natural Sciences and Technical Sciences, respectively). In this process the Department of Bioscience was divided again along the old borders resulting in the present departments of Ecoscience (ECOS) and Biology (BIOL).

### Staff categories and sections

**Fig 2:**
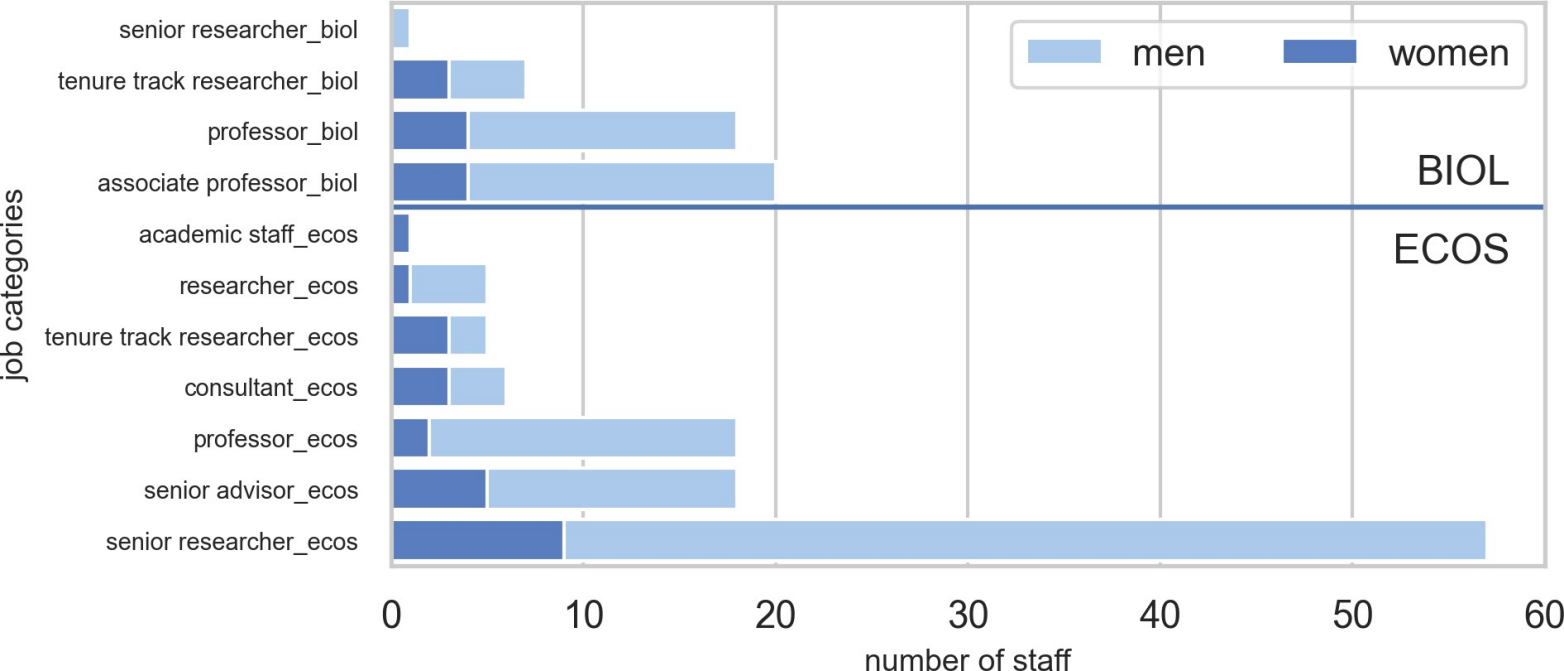
Number of permanent scientific staff per job category for BIOL and ECOS at Aarhus University. The number of women employed in each category is shown in dark blue.

The total permanent scientific staff of the two departments (per June 1^st^ 2020) was 46 for BIOL and 110 for ECOS. Differences in staff composition between the departments are shown in Fig 2. The scientific senior staff categories used at ECOS were senior advisors, senior researchers and professors. Senior staff at the BIOL were either associate professors or full professors. Common to both departments was the tenure track researcher, but ECOS also used ‘researcher’ as a junior position.

**Fig 3:**
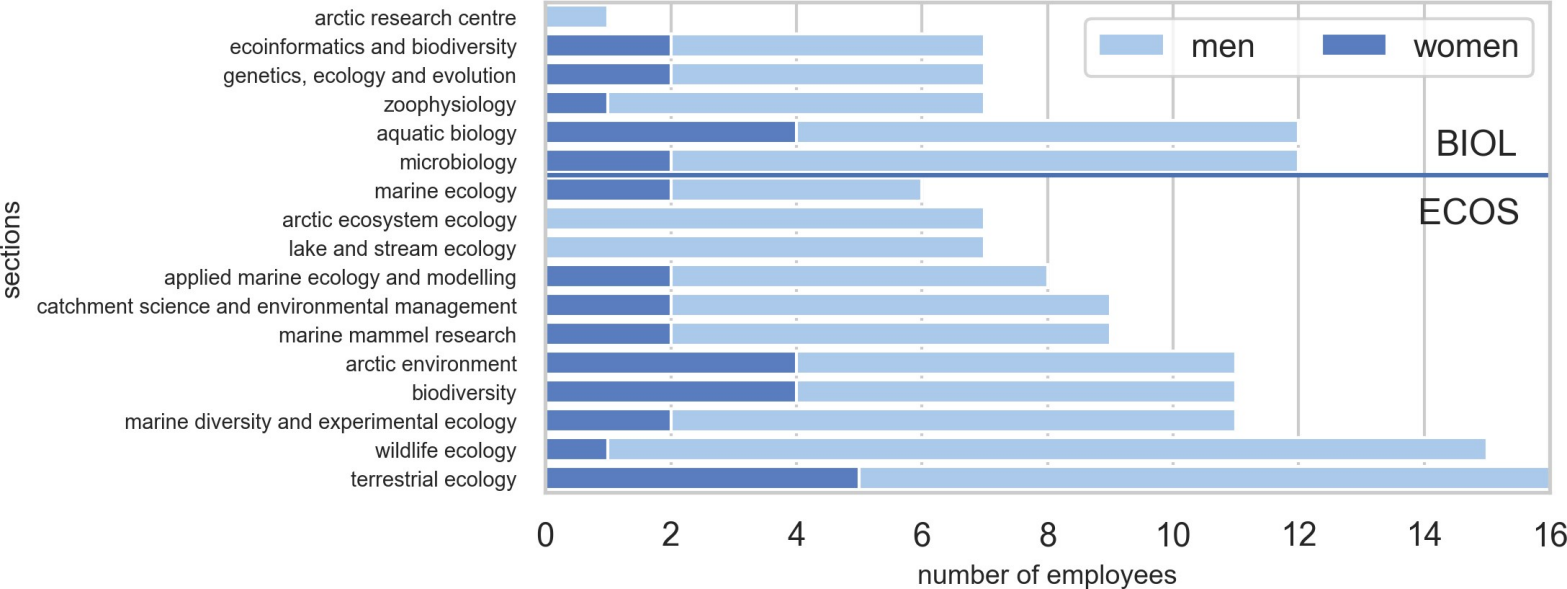
Number of permanent scientific staff per section for BIOL and ECOS at Aarhus University. The number of women employed is shown in dark blue.

Each department was subdivided into sections. ECOS had 11 sections and BIOL five sections and one research centre (Arctic Research Centre). The latter is a cross-faculty interdisciplinary research center where most participants are employed in other departments or are non-permanent and only the center coordinator is included in this study. The sections differed in size from six to 16 employees as shown in Fig 3 where section names are listed.

### Gender balance

Women constituted 23.9% and 21.8% of the permanent scientific staff at BIOL and ECOS, respectively. The average percentage across the two departments was 22.4%. The overall staff distribution and gender representation is shown in Figs 2 and 3 for job categories and sections, respectively. The Figures show that biased gender balance was less severe in the junior job category, tenure track researcher.

### Age distribution

**Fig 4:**
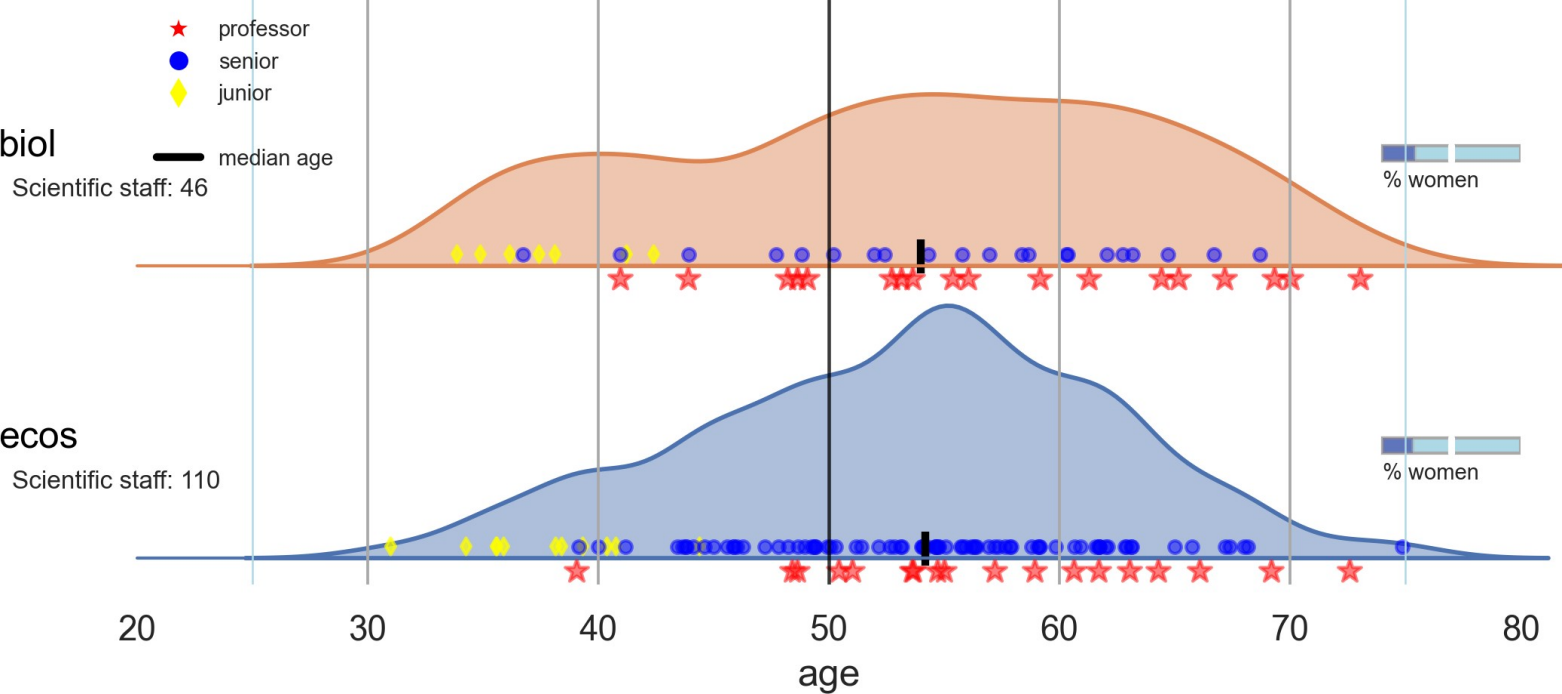
Age distribution for BIOL and ECOS at Aarhus University shown as a KDE (Kernel Density Estimator) and with the position of each individual staff member (within three basic career levels (junior, senior and professor). The median age is marked with a vertical bar on the x-axes. The bar on the right shows the percentage of women employees.

The median age of a researcher in BIOL and ECOS was 53.5 and 53.7, respectively. Fig 4 shows density curves for age distribution for each department. The figure shows that the age distribution in BIOL was relatively equal whereas the curve for ECOS showed a pronounced age peak between 50 and 60 years.

### Scientific productivity

**Fig 5:**
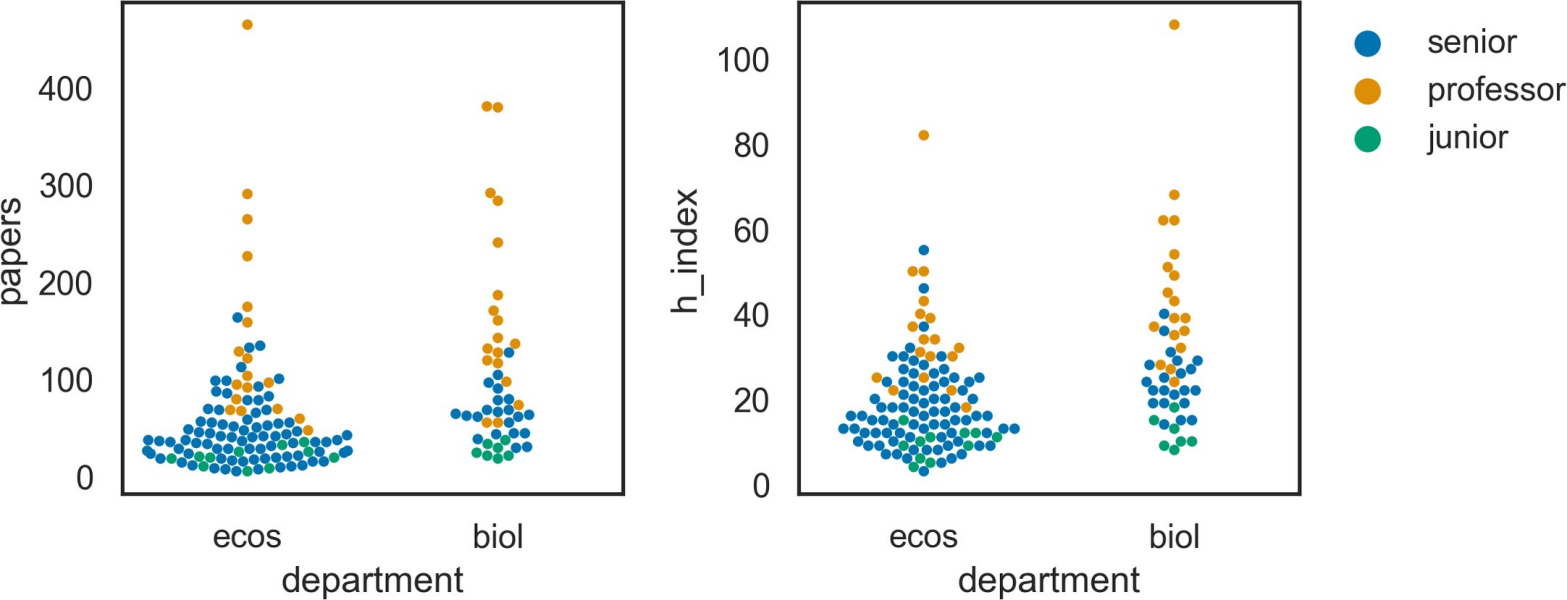
Performance indicators (Left: number of papers; right: h-index) for individual researchers at BIOL and ECOS. Each researcher is represented as a dot. The color of the dot indicates the career level of the researcher (professor, senior, and junior).

The total number of papers extracted for this study was 6,830. The average number of publications per researcher in BIOL and ECOS was 102.0 and 58.3, respectively. These numbers covered a wide range as shown in Fig 5 (left). A few researchers had more than 400 publications, but the majority had between 50 and 100 papers on their CV.

### H-index

The Hirsch- or h-index is commonly used as measure of both the productivity and citation impact of the publications of a scientist [53]. The average h-index for a researcher in ECOS was 19.7 and 31.0 in BIOL. The h-index values varied substantially as shown in Fig 5 (right) and the general pattern was similar to the distribution of papers.

### Scientific productivity and scope

**Fig 6:**
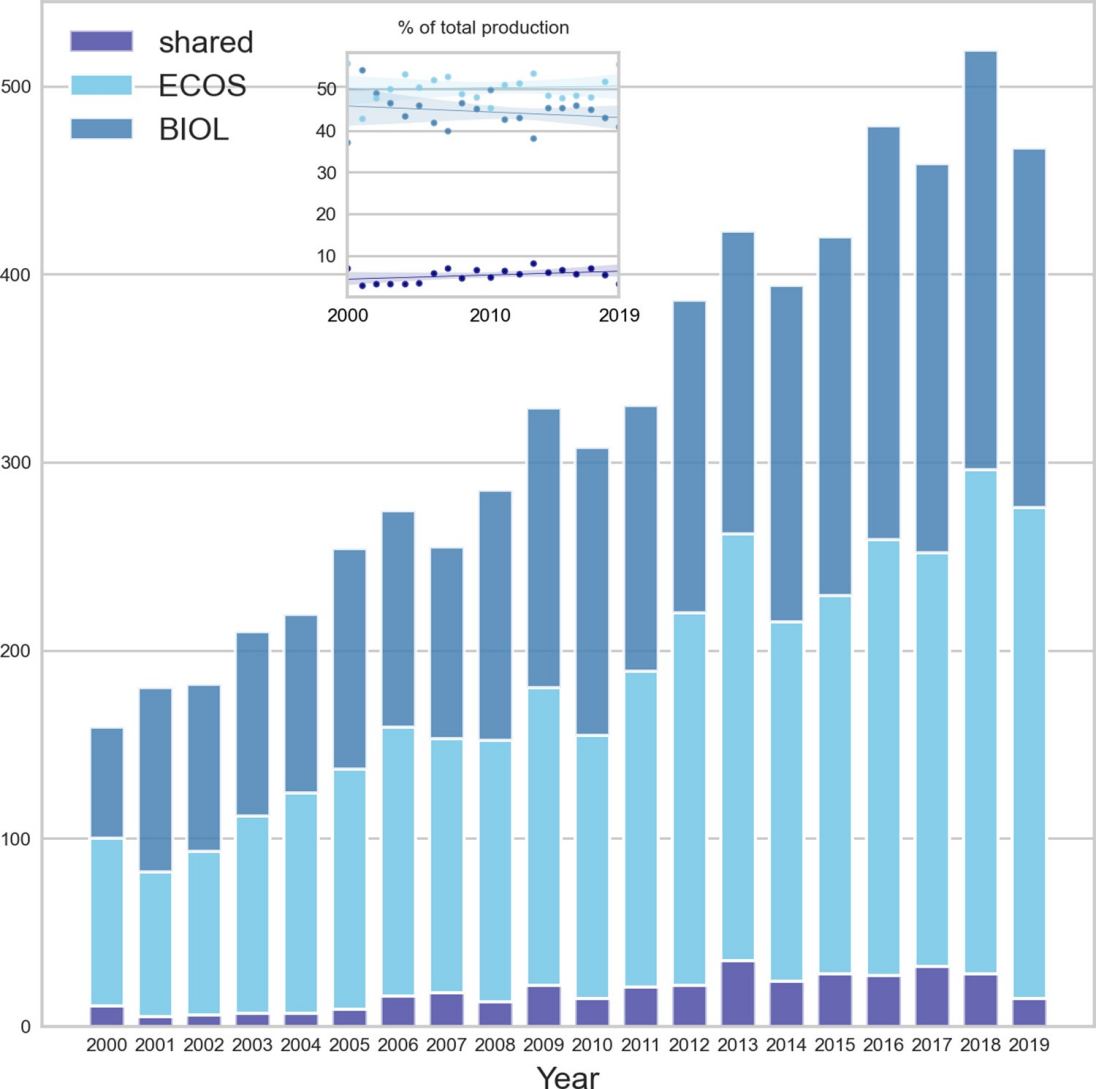
Production of papers per year for BIOL and ECOS and for shared papers with authors from both departments. The small insert shows the share of publications in percent for the two departments separately and for shared work.

The total number of papers produced per year increased from below 200 per year in 2000 to around 500 in 2019 for the two departments combined (Fig 6). Note, however, that the corpus of papers exclude publications from previous employees, which means that the actual total number of papers per year is somewhat underestimated for the first years. The share of publications from ECOS was close to 50% throughout the period. The corresponding number for BIOL was about 45% and decreased slightly over the period. Co-publications with authors from both departments constituted ca. 5% and increased somewhat from 2000–2019.

**Fig 7:**
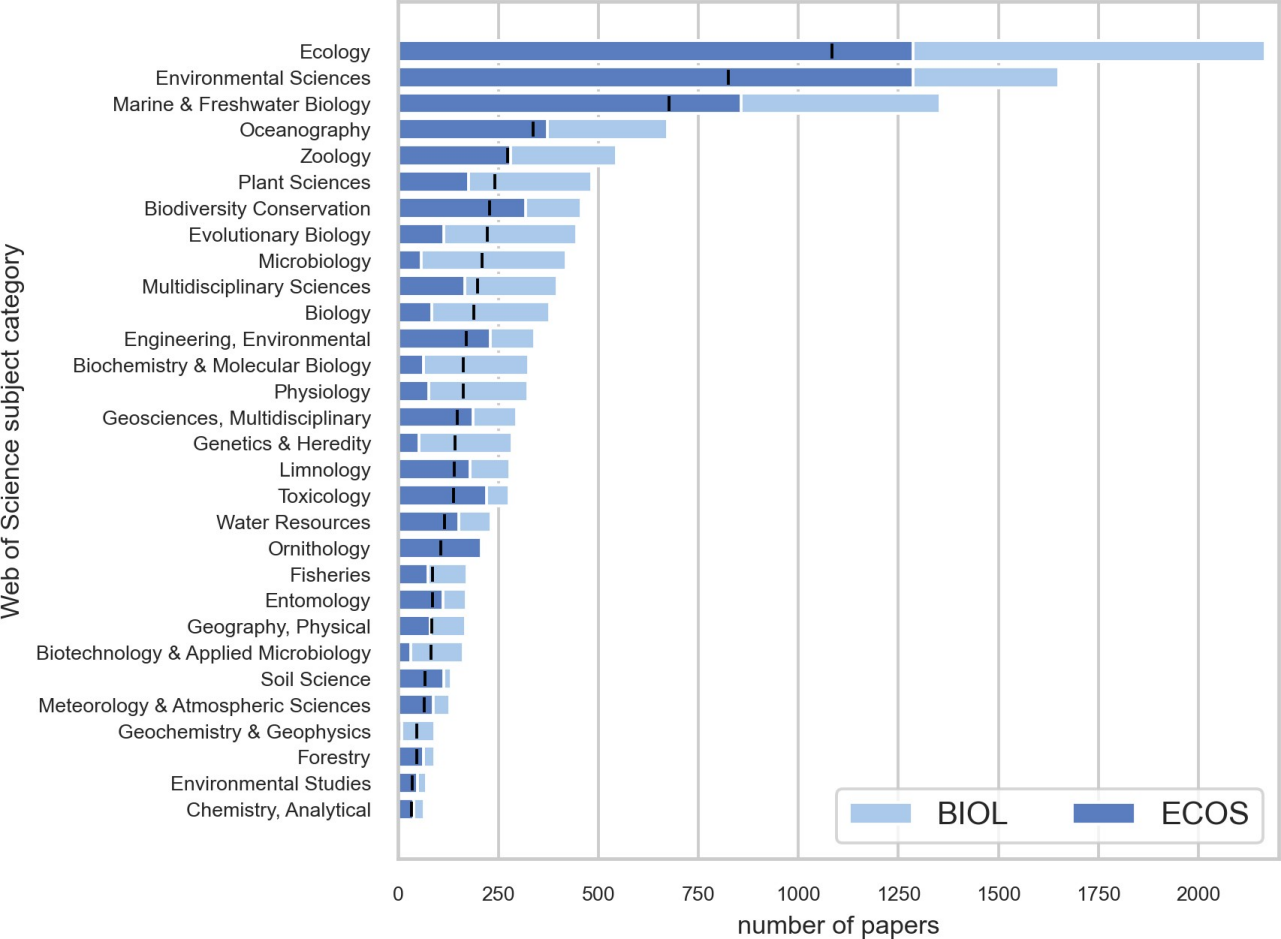
Preferred type of subject category paper chosen for publication at BIOL (light blue) and ECOS (dark blue). The black bar shows the mean value.

All journals registered in Web of Science are assigned a research subject category (for example *ecology*, *water resources*, and *biodiversity & conservation*). Not all papers in a journal do necessarily belong to that category, but a substantial part of them does and the subject category was used as a label to indicate what type of research dominated at the departmental level. Fig 7 shows the distribution of the total number of papers per subject category for BIOL and ECOS (note that some journals belong to two or more categories).

The figure shows that ECOS dominated in subject categories such as *ecology*, *environmental sciences*, *marine & freshwater biology*, *toxicology*, and *ornithology*. BIOL, on the other hand, had most publications in the categories of e.g., *plant sciences*, *evolutionary biology*, *biochemistry* & *molecular biology*.

### Distribution of keywords

**Fig 8:**
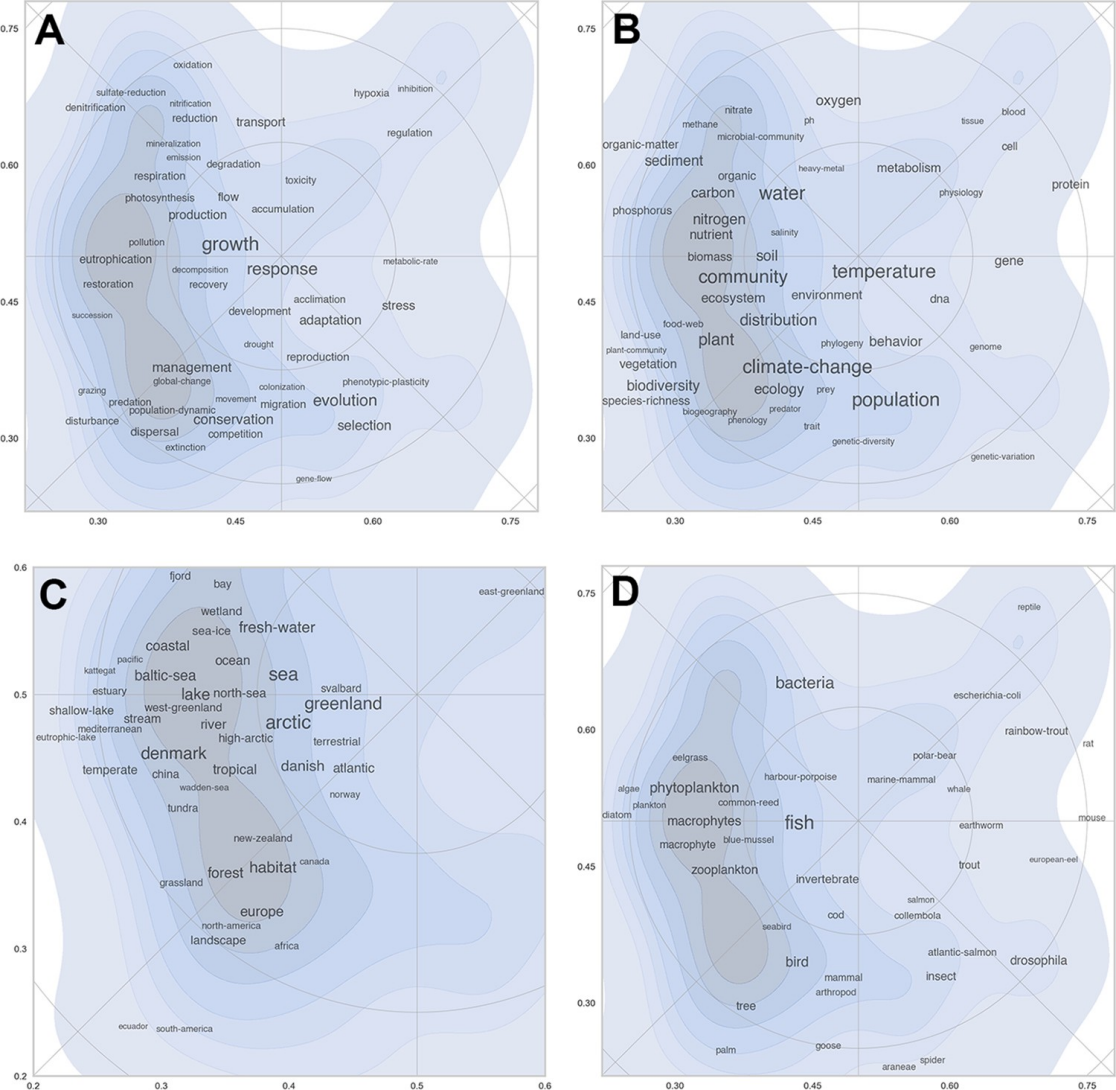
Distribution of typical keywords in four different groups. (8A), processes (8B) subjects, (8C) habitats and geography, and (8D) organisms). The purpose of the figure is to provide an understanding of the content of the map and the position of different specialties. Note that Fig 8C zooms in on a smaller part of the total map as keywords related to habitats and geography mainly were distributed in south-west quadrant of the map.

The distribution of keyword terms defined the reference map and all subsequent overlays based on it. It is important to analyze location of individual keywords in order to interpret and understand the layout and the contents of a map. As the analysis included 3,869 keywords, it was neither possible nor meaningful to show them all. Instead, to present a general impression of the map and its content, Fig 8A–8D show the position of selected keywords in four major, roughly defined groups that cover (A) *biological subje*cts, (B) *biological processes*, (C) *habitats or geography*, and finally terms that relate to (D) *taxonomy* (species or groups of organisms). The font size of keywords in the figure is proportional to the number of occurrences in the corpus. The maps showed that common keywords used in many different research specialties (e.g., *growth*, *response*, *temperature*, or *community*) were found in the center of the map. Keywords or terms that were restricted to more narrowly defined scientific domains were generally closer to the periphery of the map. Examples of such specific and domain defining keywords from Fig 8 were: *blood*, *methane*, *plant community* and *genetic variation* (subjects); *hypoxia*, *nitrification*, *pollination*, or *gene flow* (processes), *shallow lake*, *south-america* or *east-greenland* (places or habitats); or *phytoplankton*, *rainbow-trout* or *drosophila* (organisms). The distribution of keywords showed a difference between the east side of the map (experimental, lab-based research on physiology and genetics) and the west where research was more field- and ecology-oriented. This is especially evident on Fig 8C (places and habitats) where the eastern side of the map is almost empty.

Put next to each other, even simple maps as those shown in Fig 8 indicated quite accurately what kind of research was done where in the landscape. When zooming in on a specific area, for example the north-east corner of the inner circle, it is possible to extract the following keywords from the four maps: *toxicity*, *accumulation*, *metabolism*, *physiology*, *marine mammal*, *polar bear and east-greenland*. This specific area involved a research specialty within the Section of Marine Mammal Research (ECOS) that studied the relationship between climate change and the up-concentration of environmental contaminants and their impact on predatory animals in the Arctic.

**Fig 9:**
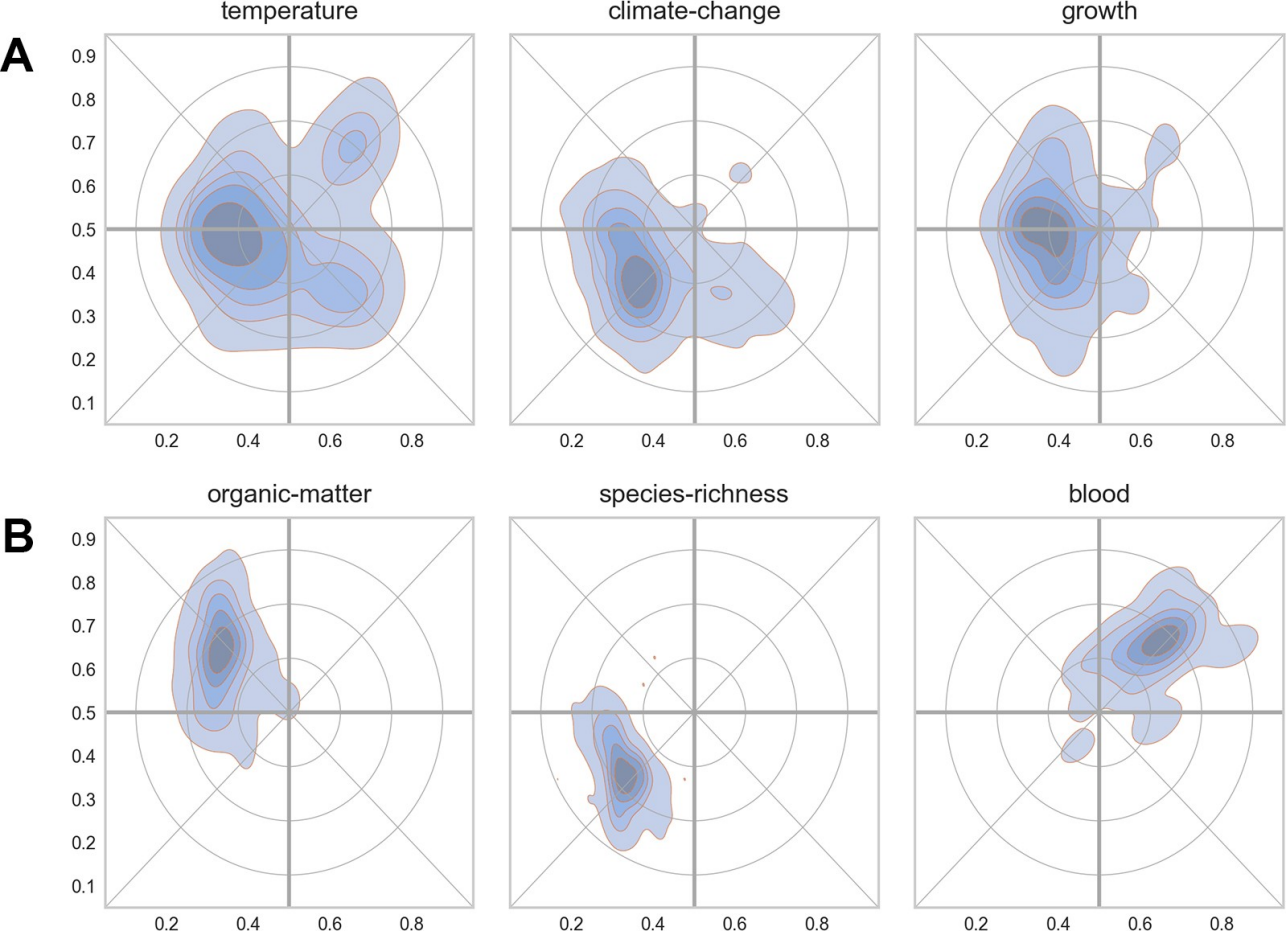
Examples of keyword distributions in the reference landscape. (9A) general keywords. (9B) more specialized keywords.

The maps in Fig 8 show the distribution of keyword terms using their x-, y-coordinates. A keyword, however, may be used in papers in various research specialties. To provide an overview of how flexible a given keyword is used, we can map all the papers in which it occurs. Fig 9 shows density plots for six keywords of varying centrality and distribution. The keyword *temperature* was found in most parts of the scientific landscape, but with a clear peak in the western part of the map where it was used in the context of aquatic and terrestrial ecology. The keyword, however, was also used in the eastern part of the map, but here in relation to physiology (towards the north-east) or to the study of genetic adaptations (towards the south-east). *Climatic change* was a trending keyword used in many different contexts and has, perhaps not surprisingly, a more or less similar distribution as the keyword *temperature*. Fig 9B shows examples of more domain specific keywords confined to much more narrow areas in the map (e.g., *organic matter*, *species richness* or *blood*).

### Distribution of scientific papers and their Web of Science subject categories

**Fig 10:**
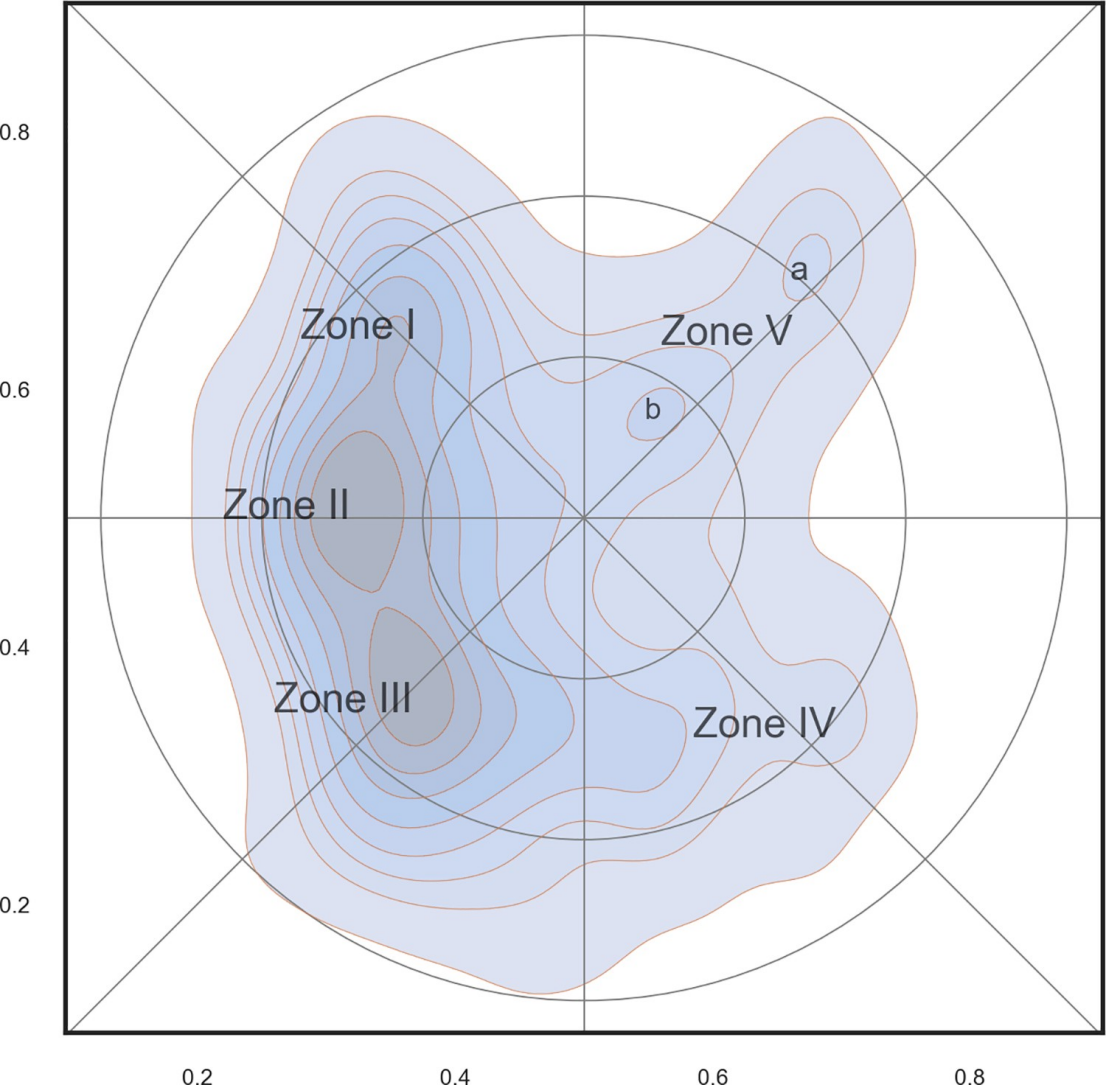
Density of papers within the science landscape as a proxy for research activity. Roman numerals from I-V indicate peaks or zones of high research activity. The labels correspond to: I: Microbiology; II: Aquatic Biology; III: Biodiversity and Terrestrial Ecology; IV: Genetics; and V: Zoophysiology and Toxicology (zone V have two minor sub-regions: a (zoophysiology) and b (toxicology).

The analyses used density of papers as a rough substitute for research activity within a specific area. This is obviously an approximation based on the assumption that publication effort and tradition are more or less even among research specialties and researchers. The map presented in Fig 10 shows some general tendencies in the research landscape. Research activity was much higher in western part of the map and formed a ridge from the top to the bottom. Along this ridge were three local peaks visible (labelled I-III). The research activity on the east side of the map was much sparser, but two smaller ridges were clearly visible stretching towards the north-east and south-east corners of the map, respectively (labelled IV-V–and ridge V with two smaller peaks–for an interpretation see below).

There were no clear boundaries between these peaks, but they may be seen as a first crude subdivision of the map into research specialties. The distribution of keywords shown in Fig 8A–8D can be used to interpret their scientific content. For example, zone I was characterized by the following keywords: *bacteria*, *bacterial communities*, *sulphate reduction*, *nitrification*, *ocean*, *sea-ice*, *sediment*, etc. These keywords point in the direction of microbiology, marine biology and nutrient cycling. Keywords found in zone V included *evolution*, *selection*, *drosophila*, *sex*, and *gene* and are clearly related to the broad field of genetics.

Another way of visualizing the distribution of research specialties in the landscape is to make use of the research categories in Web of Science where all journals belong to one or several subject categories such as *Ecology*, *Biology*, *Conservation Ecology*, *Toxicology*, etc. These categories may be broad and very general (such as *Ecology* or *Biology*), or sometimes very specific (*Ornithology*). Subject categories are used to label journals and some papers may end up in a category they do not fit in. Even so, when applied to a large number of papers, the categories provide a good indication of what kind of research characterizes which part of the landscape.

**Fig 11:**
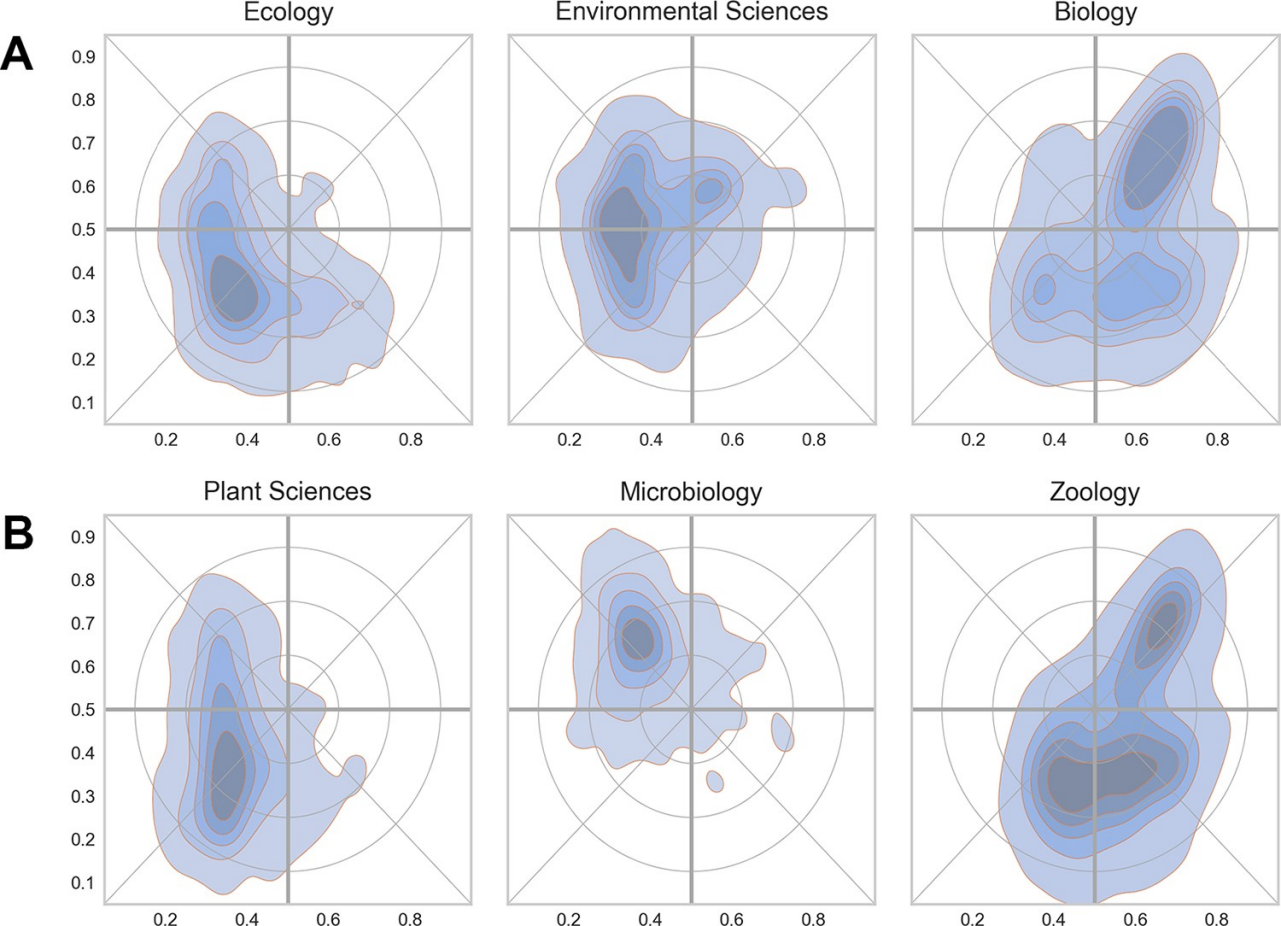
Examples of narrow (11A) and wide (11B) Web of Science Subject Categories in the reference landscapes. The color of the shading is not uniform across the figure, but is relative to the category in question.

Fig 11 shows density maps for three relatively broad Web of Science categories. The categories of *Ecology* and *Environmental Sciences* had their major distribution towards the western part of the map, whereas *Biology* had the highest density on the eastern side. Some categories were narrower and confined to small areas and were therefore more precise indicators of scientific activity. Fig 11 shows the density profiles for *Microbiology*, *Conservation Ecology* and *Evolutionary Biology* with centers of distribution in three of the four major quadrants in the map (north-west, south-west and south-east, respectively).

**Fig 12:**
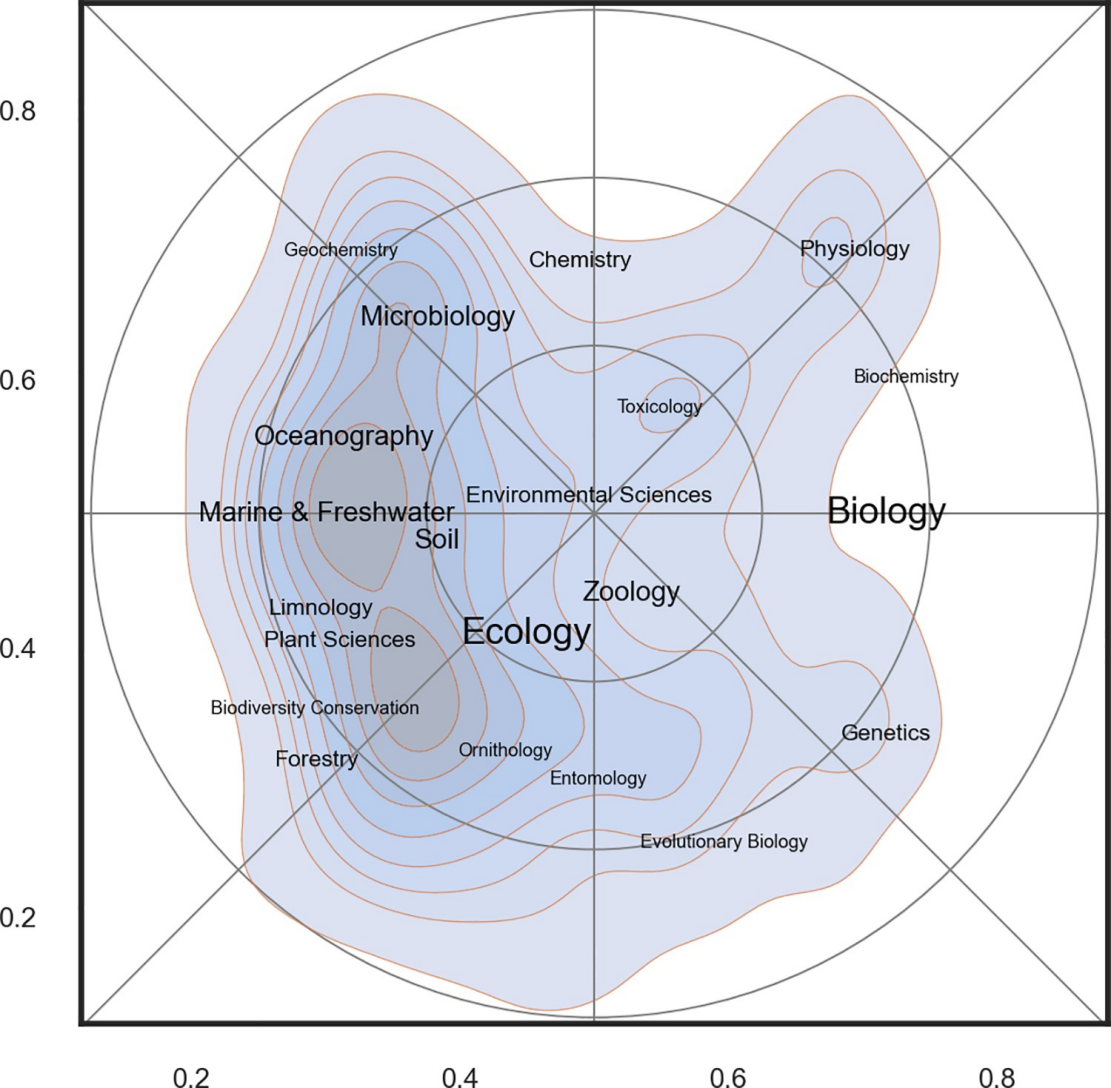
Basic science map for biology at Aarhus University. The shading corresponds to density of papers and is a proxy for research activity. Labels are Web of Science subject categories placed in the center of their distribution. The font size is proportional to the importance of the category (see also Fig 7).

Fig 12 shows the most commonly used subject categories for papers produced by ECOS and BIOL. A label is placed in the center of each category, and the size of the label text reflects the number of papers within it. The map confirms the findings in the previous paragraphs and makes it possible to assign a label to each of the five zones shown in Fig 10:

I: Microbiology; II: Aquatic Biology; III: Biodiversity and Terrestrial Ecology; IV: Genetics; and V: Zoophysiology and Toxicology (zone V may has two sub-regions: a (zoophysiology) and b (toxicology)). These labels will be used in some of the maps shown below and serve as landmarks.

### Profile maps for departments

Both departments work within the general field of biology, but there are noticeable differences in research focus.

**Fig 13:**
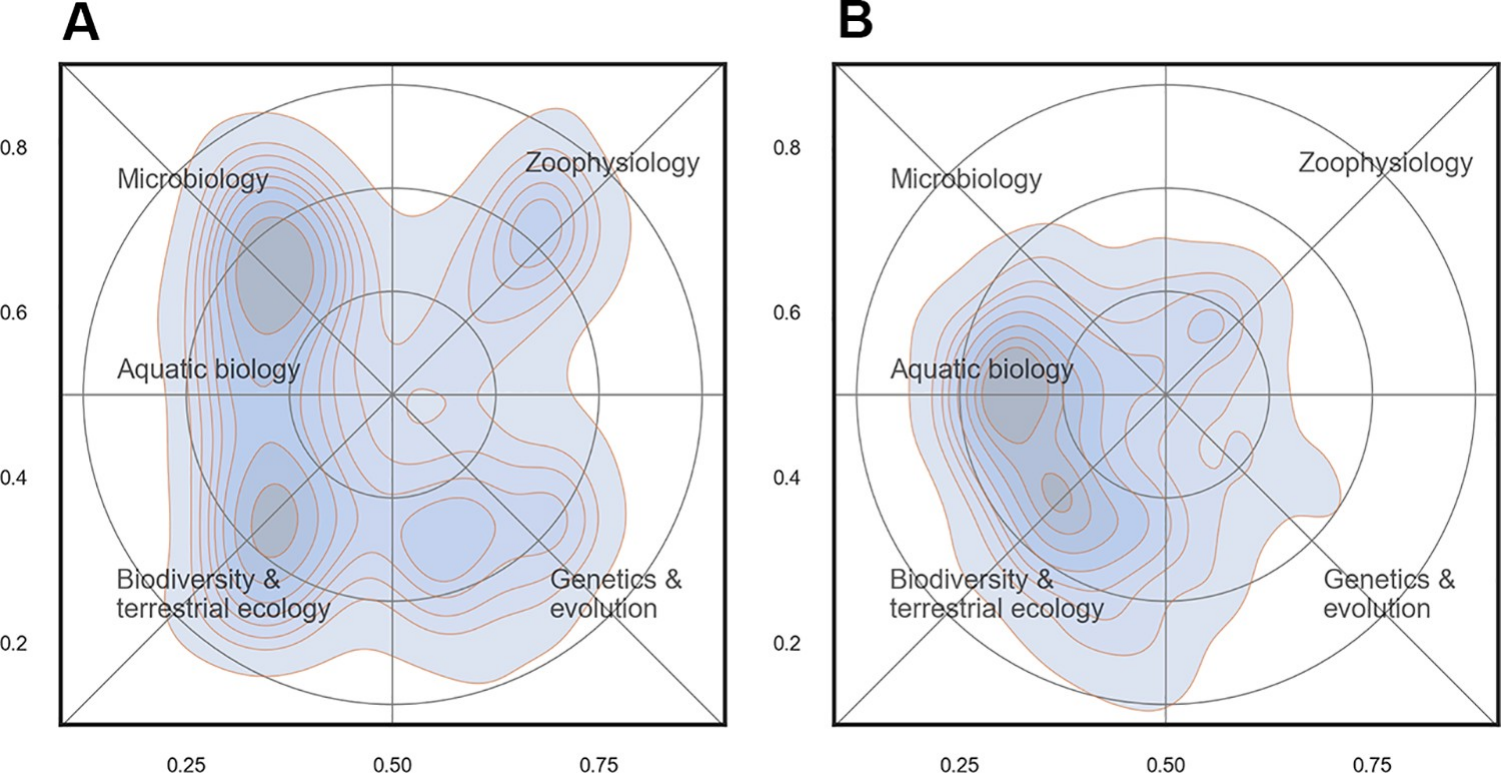
Profile maps for ECOS (13A) and BIOL (13B). Contour plots show the density of scientific papers in the landscape. The most important Web of Science subject categories are included as place names. Annotations in blue summarize the content of the map.

Profile maps for each department are shown in Fig 13. BIOL had four distinct peaks of activity, although not all of equal height or density. The department was generally strongest (or most active) on the western side of the map where mainly the fields of microbiology and terrestrial ecology were prominent, but there were also two distinct peaks for zoophysiology and genetics and evolution towards the east. The corresponding map for ECOS shows that the research activities were much more concentrated and predominantly towards the west with a focus on terrestrial- and aquatic ecology.

The maps reveal that the departments were different in a number of ways: the scientific breadth was broader in BIOL compared to ECOS (more activity towards the periphery of the map), and clusters of research were more distinct in BIOL.

### Profile maps for research sections and groups

The two departments were each subdivided into a number of sections each led by a Head of Section. ECOS had 11 sections and BIOL had five sections and one interdisciplinary research center. Sections varied in size and their composition reflected research specialty, history (former organizational units) and the major tasks carried out in the section.

**Fig 14:**
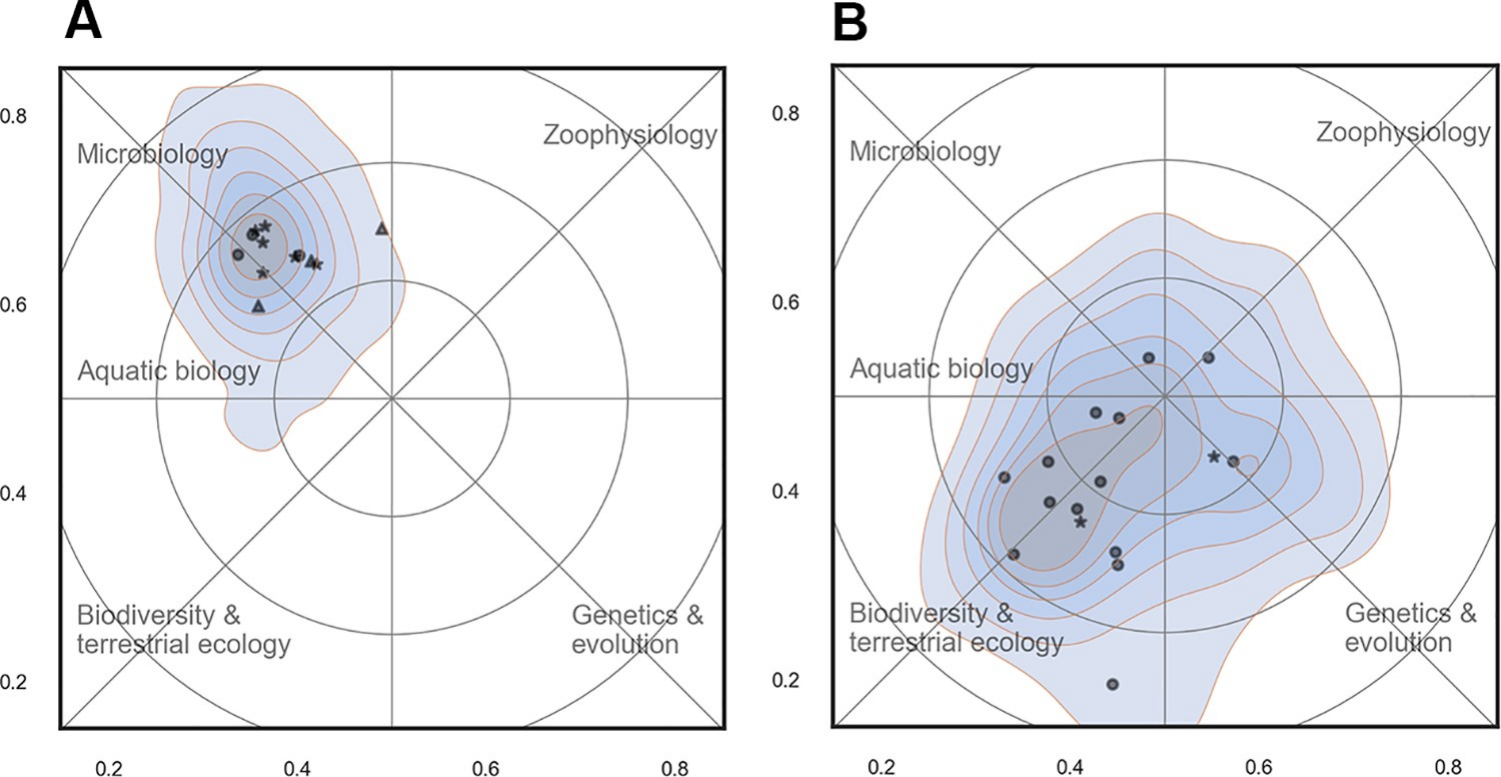
Profile maps for the sections of Microbiology from BIOL (14A) and Terrestrial Ecology from ECOS (14B). Contour plots show the density of scientific papers in the landscape. The most important Web of Science subject categories are included as place names. Annotations in blue summarize the content of the map.

**Fig 15:**
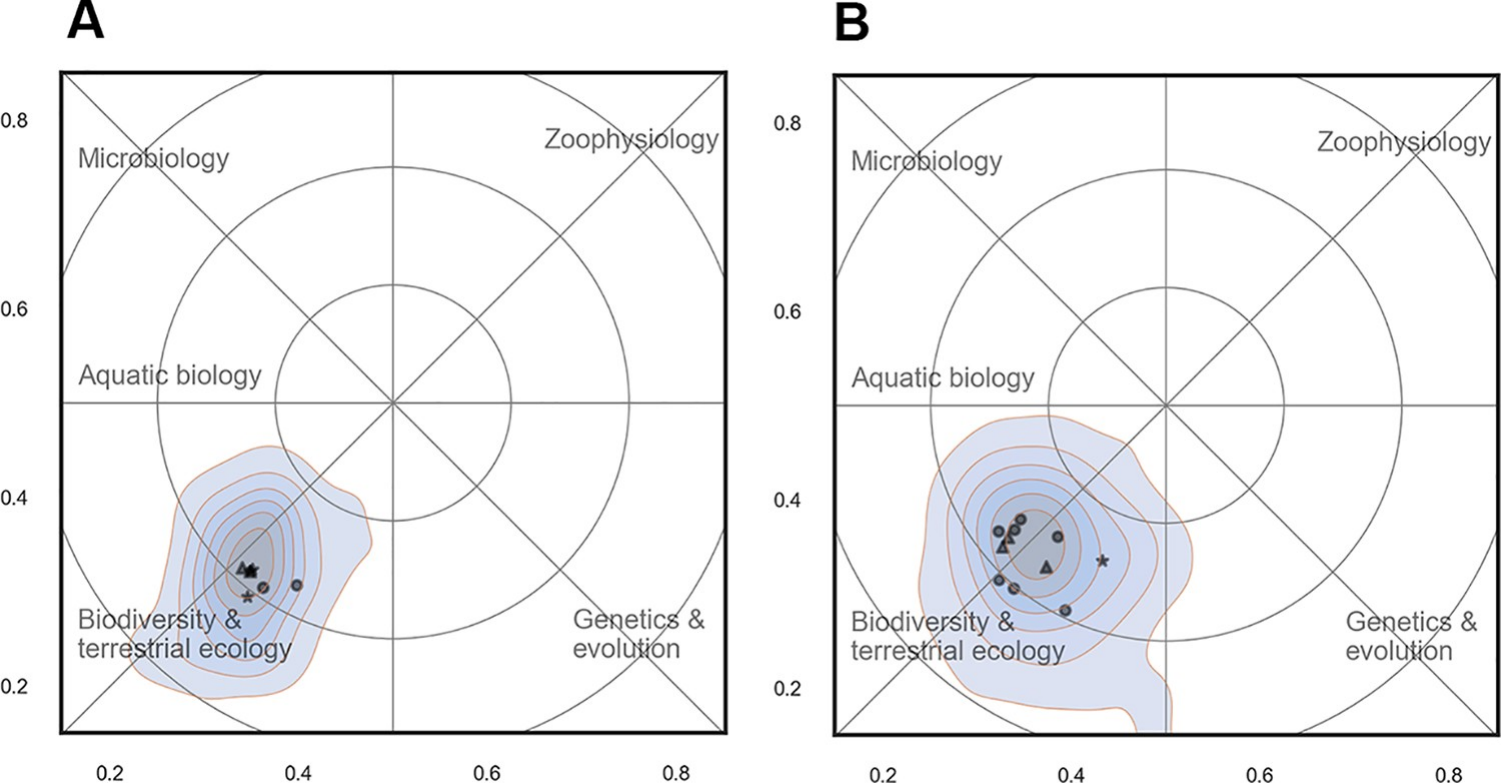
Profile maps for the sections of Eco-informatics and Biodiversity from BIOL (15A) and Biodiversity from ECOS (15B). Contour plots show the density of scientific papers in the landscape. The most important Web of Science subject categories are included as place names. Annotations in blue summarize the content of the map.

Fig 14 shows two sections that differed both in scientific focus and in size. The map on the west side of Fig 14 shows the section of Microbiology (BIOL) characterized by a rather narrow research focus. The map on the right side of the figure shows the section of Terrestrial Ecology (ECOS) with a completely different research focus and a much wider scope. The latter section was recently formed by merging two smaller sections (Plant-insect Interactions and Eco-toxicology and Soil Organisms). This is not obvious from the current name, and a good example of how a profile map in a glimpse may show what kind of research takes place within a section and how it relates to other units. The section of Terrestrial Ecology also had a substantial overlap with the sections of Eco-informatics and Biodiversity (see Fig 15).

In other cases, sections may cover almost the same research specialty. Fig 15 below shows an example of this, where the sections of Eco-informatics and Biodiversity (BIOL) and Biodiversity (ECOS) were almost indistinguishable in terms of scientific content. This doesn’t necessarily mean that the sections do the exact same research, but there was at least a substantial overlap and the differences were too small to be visible using the resolution of the current reference map.

**Fig 16:**
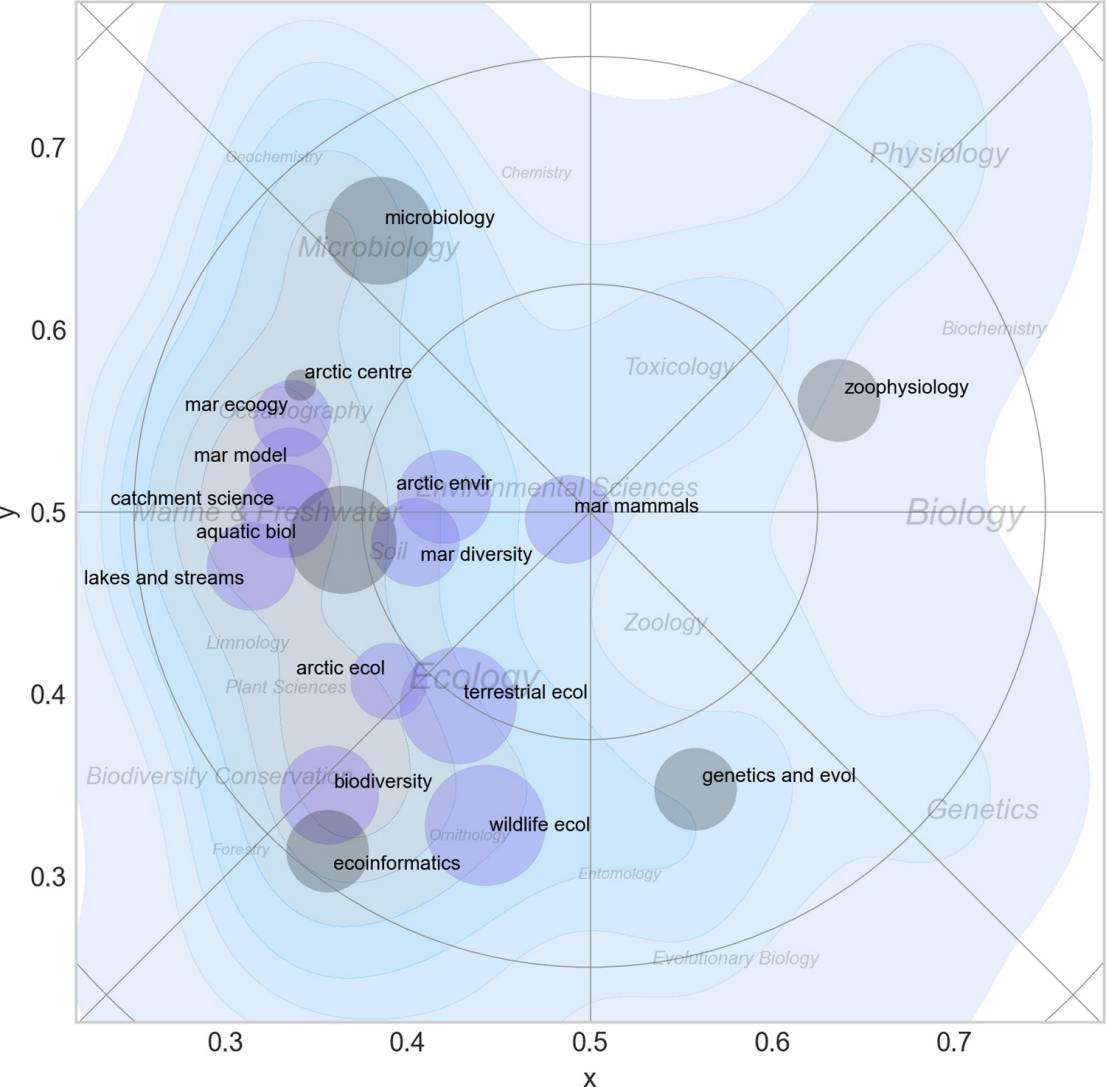
All sections in ECOS and BIOL and their distribution in the scientific landscape. Each section is represented by a bubble with a diameter corresponding to its number of permanent scientific staff. Green = BIOL. blue = ECOS. Contour plots show the density of scientific papers in the landscape. The most important Web of Science subject categories are included as place names (see also Fig 12).

Fig 16 shows the distribution of all sections in the two departments. Each section is represented by a bubble placed in the center of its distribution. The size of the bubble reflects the number of permanent scientific staff in each section.

Fig 16 confirms the differences noted above between the departments. Sections from BIOL were in general more specialized and distinct than sections in ECOS that had a relatively high degree of overlap. This is especially prominent in the western side of map, where there was a high number of sections with overlapping boundaries in the marine and aquatic research domain.

### Researchers in the landscape

Individual researchers were represented in the scientific landscape as a continuous field showing the density of papers produced or with a single symbol placed in the center of the point cloud. The former representation is useful when we are interested in the actual scientific width and breadth of a researcher; or to see how his or her scientific focus may have evolved and changed over time. In most cases, however, it is sufficient to represent researchers with a single symbol; especially when the aim is to produce an uncluttered map with many researchers.

**Fig 17:**
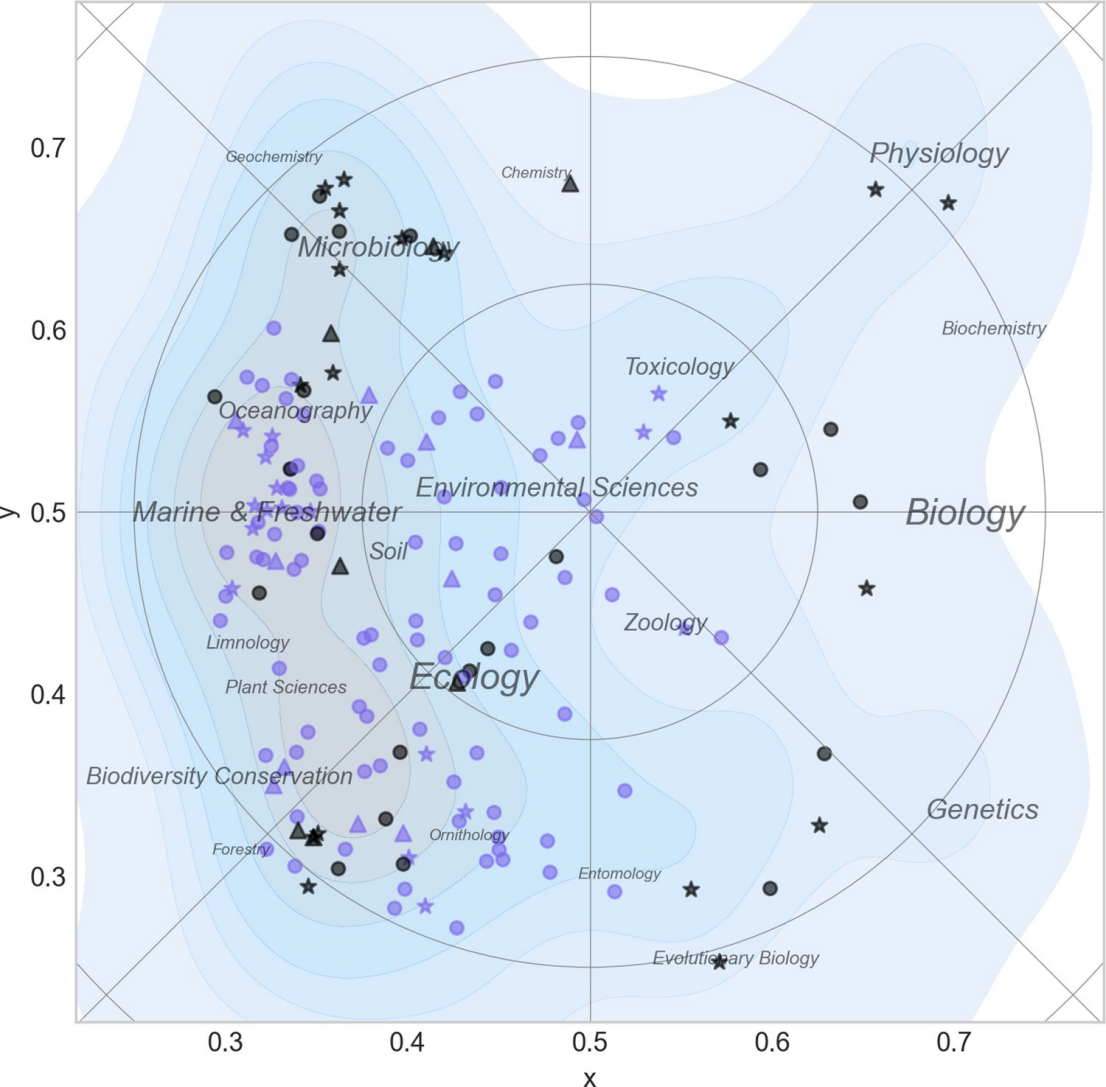
Distribution of permanent scientific staff from ECOS and BIOL. Each researcher is represented by a marker (stars = professors, circles = senior staff; triangles = junior staff) and a color (green = BIOL. blue = ECOS. Contour plots show the density of all papers in the landscape. The most important Web of Science subject categories are included as place names (see also Fig 12).

Fig 17 shows the distribution of all permanent staff at the two departments where some general tendencies are noticeable. Researchers from BIOL were often closer to the periphery and thus more specialized. They were, furthermore, scattered throughout the map and covered most of the research specialties within the landscape. Researchers from ECOS, on the other hand, were generally confined to a narrower scientific realm (mainly ecology) and tended to be closer (scientifically) to each other. The figure also shows the distribution of professors, senior- and junior staff. Most areas had a relatively equal distribution of the different categories, but some areas, e.g., zoophysiology had several professors, but lacked junior- and senior staff. Other areas such as microbiology, aquatic ecology and terrestrial ecology had equally high concentration of all staff categories.

### Age- and gender distribution in the landscape

Science maps may also be used to visualize structural patterns in the organization.

**Fig 18:**
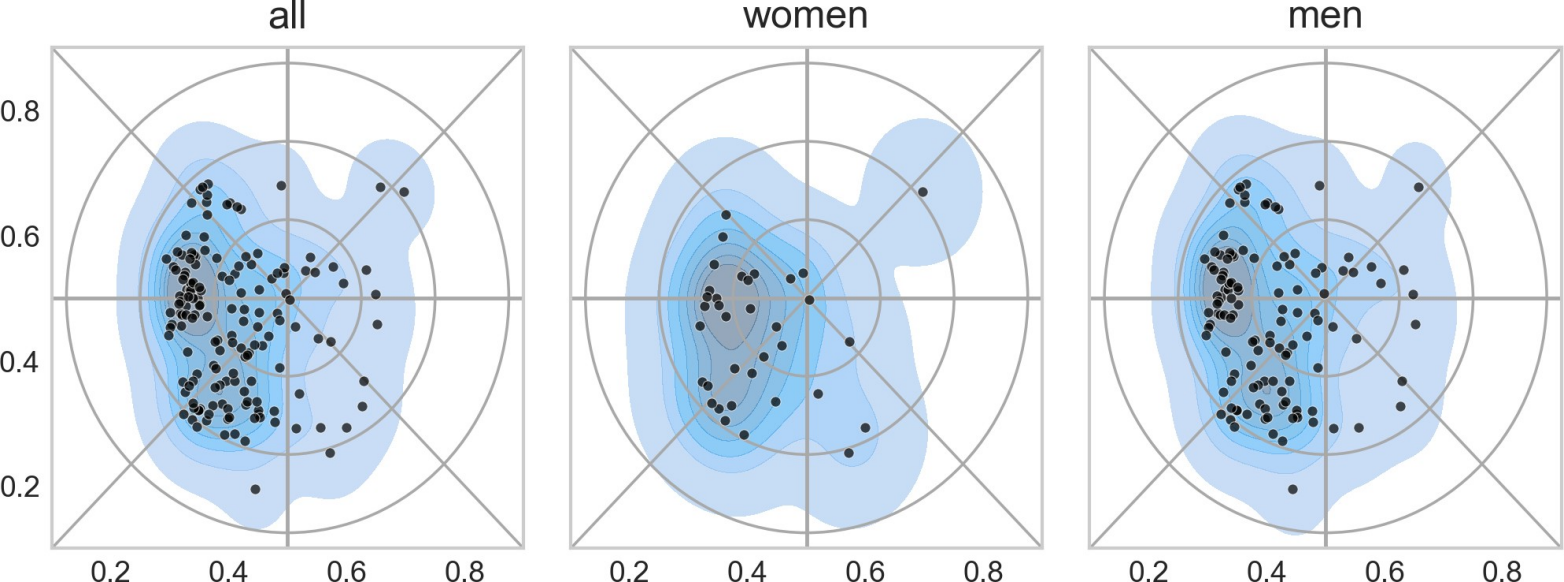
Distribution and density of staff in the landscape (left: all; middle: female; and right: male).

Fig 18 confirms that the distribution of gender at the department was skewed (women constituted one fourth of the permanent staff–see also Figs 2 and 3), but the distribution and density patterns were more or less the same for men and women across the landscape. Gender bias, in other words, is a general problem and not confined to specific areas.

**Fig 19:**
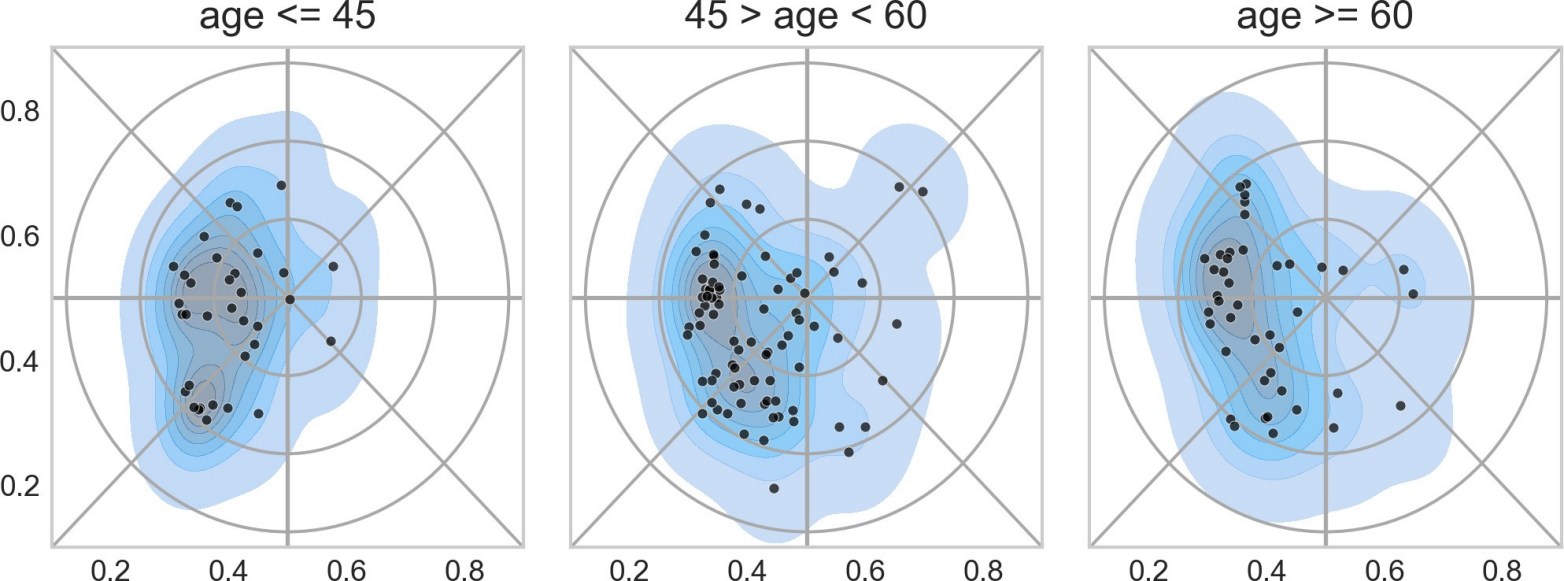
Distribution of three age groups in the landscape (left: age below 45; middle: age between 45 and 60; and right: age over 60.

Fig 19 shows that recruitment of permanent staff predominantly had taken place in the western part of the map. This indicates a need for recruitment in the classic disciplines of zoophysiology and genetics. Fig 19 also shows that younger researchers tended to group in two clusters. One was the south-west part of the map (biodiversity and terrestrial ecology) and one near the center of the map (aquatic ecology in the wider sense.)

## Discussion

This study explores how ‘soft’ information in the form of science maps (basically the distribution of subjects, papers and people and in a scientific cognitive space) may be combined with traditional statistics and performance indicators to drive and inspire strategic thinking. Descriptive data commonly used for strategy development (such as current organization, staff number, positions, bibliometric score, gender, age, etc.), is very valuable and planning is not possible without it. However, it may often restrict creativity because existing structures act as a mind blocker that works against innovative thinking. This paper uses a science mapping approach to map what kind of research is done where, who are involved, and try to shed some light on where there are potentials not currently utilized or where overlap or competition might occur. The method is based on extracted keyword terms (what authors actually study and write about) and not on potentially biased assumptions on what individual researchers and existing groups do or should do. It is important to remember that every map is an abstraction. The final layout depends on many choices in the mapping process and is not totally value-free. However, the maps are ‘mechanically objective’ in the sense that a map can be reproduced by other researchers following the same methodology. A clear and concise documentation of the workflow is therefore necessary to secure transparency, facilitate the interpretation and to avoid that science maps become black boxes [25].

### Assessing the general efficacy of the reference map

A good reference map is based on solid data and methods and should be applicable for practical purposes [25]. In a regular cartographic map, users may pinpoint their own position and find features or areas of interest. Science maps are much more abstract than geospatial maps that represent a physical reality, but studies show that users (when properly guided) are able to understand and navigate them [54].

Some features of the reference map presented in this study differ from a regular geospatial map and require clarification:

1. The position of a research unit (keywords, papers, authors, or groups of authors) in relation to the center/periphery of the map is important. Generally, units that are close to the periphery, tend to be more specialized or (less interdisciplinary in case of groups) than units close to the center.
2. Units closer to each other are more similar than units far apart. This is the so called first principle of geography: “everything is related to everything else, but near things are more related than distant things” [55]. Difference in distances, however, is not the same everywhere in the map. The density of keywords and scientific activity are much higher and concentrated in the west side of the reference map, where the degree of change is relatively larger over shorter distances than in the east side of the map. This is like comparing a mountain area to a topographically flat region. In the former, many different altitudinal zones exist within short distances, whereas it is necessary to travel greater distances to experience different conditions in the flatter landscapes. However, the maps still provide a strong sense of distance and direction and most researchers will without difficulty recognize their neighborhood and the researchers they are associated with in the map.
3. The continuous field maps created in this paper are fuzzy in the sense that there are no fixed borders of units. The delimitations are not absolute, but relative and based on a certain measure of subjectivity.

The corpus of articles used in this study is the total production of papers co-authored by the current staff. This is a small sample and does not represent the full field of biology. A closer look at the map reveals that important subfields of biology are missing or underrepresented. This is the case for, e.g., molecular biology, cell biology and biochemistry. These important research fields are present at Aarhus University, but belong to the Department of Molecular Biology. Other subfields have been more prominent earlier, but are less important today. Examples of this are the classic sub-disciplines of mycology and lichenology that have almost disappeared, but also traditional fields of botany, zoology and especially morphology and taxonomy are much less important today than they used to be. A larger and more representative sample could be drawn from Web of Science to create a reference map with a larger cognitive extent and scale. There is, however, always a trade-off of between accuracy and overview and the choice of correct scale should be considered carefully [49]. This is analogous to the geospatial map where large scale maps of the globe or continents provide excellent overview, but are unsuitable for, e.g., detailed direction-finding between two localities. Since science maps do not refer to a physical reality it’s not straightforward to zoom in and out as the distribution of keyword terms must be recalculated at every new scale and their relative positions may shift as a result.

### What insights for strategic thinking do the analyses provide?

The current study shows that it is feasible to extract a wealth of information about a research unit from various online sources and convert data into visual statistics and maps. The results, which are discussed below, show both differences and similarities between BIOL and ECOS.

#### Statistics and performance indicators

*Size of departments and use of positions*. The total tenured staff of researchers at BIOL and ECOS was 156 (46 and 110 for BIOL and ECOS, respectively). In comparison, the Department of Biology at Copenhagen University has a tenured staff of 90 associate and full professors (http://e-pages.dk/ku/1404/) which makes Aarhus the largest biology research cluster in Denmark. The difference in size between ECOS and BIOL would be less pronounced if the study had included short-term scientific staff (mainly postdocs and PhD-students of which BIOL has a larger proportion. The use of job titles reflects the main difference between the two departments: BIOL is a traditional university department with focus on research and teaching whereas ECOS does applied research and advisory work especially for the Danish authorities.

*Author impact*. The total number of papers produced by the two departments has increased from about 175 per year in 2000 to 475 in 2019. Note, however, that only papers by the current scientific staff were included which means that the actual number of papers in the beginning of the period is somewhat underestimated. Each department publishes approximately the same number of papers and the average number per tenured staff member is twice as high in BIOL as in ECOS. The main reason for this is probably that many researchers ECOS has advisory work as their main task and that BIOL has more of PhD-students and postdocs. There are several ways to measure an author’s productivity and impact. Among the most commonly used are total number of papers, number of citations and the h-index, but bibliometric indicators should be used cautiously and first after addressing their limitations and biases [14]. The h-index, for example, is commonly used as a measure of an author’s scientific impact, but uncritical use of it should be avoided for several reasons. First, the index is biased against young researchers as it is strongly related to the time a publication has existed. Second, it is limited by the total number of publications and thus disfavors scientists with a small number of highly cited paper. It remains, nonetheless, a common measure of research performance [14]. Similarly, the number of papers and especially number of citations depend on the research discipline and cannot be compared directly without some form of normalization [14]. Fig 5 shows the distributions of performance indicators for each department. The distributions are almost Paretian in shape, where a small group of researchers are very productive and have high h-index scores. In general, BIOL has higher values than ECOS reflecting the difference between a traditional university department with focus on high impact science and a research institution targeted at advisory work where scientific reports often are a major product.

*Gender equality*. The share of women in both departments was low (between 20.2 and 23.9% for the two departments, respectively (see Figs 2 and 3). This is far from the declared goal of equal gender balance and should be addressed specifically in strategy development.

*Age distribution*. The median age was close to 54 for both departments, but their age profiles were very different (Fig 4). BIOL had an even age distribution profile, whereas the age distribution in ECOS showed a distinct age peak close to 55 year which means that 50% of the staff is less than 15 years from retirement making it necessary to start plans for recruitment in the near future to secure a smooth transition without loss of knowledge.

*Team size*. A section is the main sub-departmental unit and is where most researchers feel at home, where their closest colleagues are, and thus the unit with which they identify. Research groups of this size are very important for the everyday work and for the social life within a department, and well-functioning sections are very important for the collective performance and health of a department. The size of sections varied between 6 and 16 employees, but here only permanent staff was included. Many sections, especially at BIOL, has a substantial number of PhD-students and postdocs that contribute actively to the production of scientific papers. The optimal size and composition of a research team have been studied extensively. One study found that research impact grows with increasing team size, whereas research novelty had a U-shaped relationship with team size [56]. Other studies show that research creativity was associated with relatively small group size with complementary skills, stable research grants, and good, facilitating leadership [57]. Collaborative research, on the other hand, often results in higher citation rates [58], because most scientific questions are complex and are best dealt with in a team of researchers with complementary expertise. A study of the effects of team size on performance on complex tasks [59] found that individuals in teams applied lower overall efforts, but collaborated more with increasing team size. In general, teams outperformed a similar number of individuals. Hence, there is no ‘best’ team size or composition, but the issue is important for the dynamics of a research team and should be analyzed carefully case by case to tackle the specific aims of the section.

#### Science mapping

*Research activity*. The scientific activity in the landscape was not equally distributed and the majority of research took place at the west side of the map (Fig 12). This area may broadly may be labelled ‘ecology’ and constituted a wide ridge running from north to south. When comparing the two departments (Fig 13), it is clear that there are overlapping areas, but also distinctive differences in scientific focus: BIOL covered a wider part of the map and more research specialties and had five rather distinct peaks in the landscape: biodiversity (mainly terrestrial), aquatic ecology, microbiology, zoophysiology and genetics & evolution). These peaks also form the basis for the division of the departments into sections. In ECOS, on the other hand, research effort were much more concentrated towards the west of the map that is associated with ecology. As discussed above, the reference map generated for this study does not span the full width of the research field of biology. Even though the actual extent of the realm of biology is a matter of subjectivity, it is clear that important parts of the full biological landscape were not represented very well in the sample drawn for this study.

*Scientific overlap between sections*. In BIOL particularly, sections were spread across the landscape and clearly separated. In ECOS, on the other hand, the separation was less clear and with much more overlap. This is partly explained by the history of the two departments, where BIOL is a merger of five departments from the central campus at Aarhus University (actually six, as zoology and genetics were fused earlier). The staff at ECOS, however, is sited at three geographically separate locations and sections are confined within these locations. This has resulted in sections with scientific overlap where expertise in a given scientific area is spread across locations (marine ecology being a good example–see Fig 16).

In any case, the overlap of research groups or lack of coverage in other parts of the landscape should be considered in the strategic process. Sections from ECOS and BIOL (and within ECOS) have overlapping research and competences, which creates a potential for collaboration and sharing of equipment and facilities, but also for competition for the same funding, students and potential candidates during recruitment. The goals and plans for sections in these zones should therefore be considered carefully during the strategic process. This could also be practiced on higher organizational levels between departments at different universities looking for possibilities for collaboration or joint ventures in teaching and advisory work.

*Distribution of researchers and their age & gender*. Both departments had biased gender distribution with few women and a skewed age distribution towards the older age groups. An overlay of staff data on the science map (Figs 18 and 19) showed that the problems of gender bias and an ageing staff were more or less evenly distributed and not confined to any specific research specialty.

A closer inspection of data and maps may evidently draw attention to other problems or opportunities. A few of the most obvious questions to consider for the field of biology at Aarhus University are listed below:

- The size of sections varies considerably and often for historical reasons. Some sections have considerable overlap in the science landscape. Is this the optimal structure or are there better ways to organize sections that may enhance productivity and creativity?
- Both departments have skewed gender and age distributions. Which parts of the science landscape are in need of new blood and which parts are most likely to attract young researchers?
- ECOS and BIOL focus on research & teaching and research & advisory work, respectively. Are there areas in the map with overlapping research expertise not used for teaching or for advisory work?
- The science map and the projected activity shows the current research focus. Is the width and depth of contemporary biology covered or are there specific areas that need to be included or advanced?

### Strategy development–top-down or bottom-up?

Strategy development is usually understood as a top-down process that is initiated and organized by the faculty- or university management. The academic staff often perceive this type of formal strategy work as rigid and controlled which leads to a lack ownership and commitment. Researchers typically pursue their own research goals and interests, and the overall direction of research is very much determined by the success of individuals and their ability to attract funding, students and co-workers. Under these circumstances strategy tends to evolve from the bottom and up. This can be considered a ‘grass root’ model for strategy planning. It has been termed ‘adhocracy’ [60] and can be seen as an emergent rather than an intended strategy development. In this interpretation, strategy and plans may evolve wherever people have the capacity to learn and where there is room and resources to support them. Emerging patterns may finally become organizational when they have grown in size, are collective and creep into the organization. At most universities, reality lies somewhere in between and it could be argued that the most important task of management is to support emerging strategies while maintaining overview and keeping the common good of the organization in mind. The intention of this paper is to explore how the use of science mapping may improve and inspire intended as well as emergent strategy processes. Science mapping offers alternative ways of viewing the contents and dynamics of a scientific research domain and thus supplements the performance indicators and data normally used as input to a strategy process. Science maps may appeal to the individual researcher, as well as research managers on higher administrative levels.

### Strategy development as a board game

Skov et al. [61] suggested to use a classical strategy board game as an analogy for science maps to visualize and facilitate strategic thinking. The board (or in this case the science map) sets up the world in which the strategy development operates. The board may show our own positions (‘territory’) and where other groups are (potential partners or rivals). This is useful information when deciding which of all the possible future moves would be the wisest. Map reading is two-dimensional and visually stimulate pattern recognition in ways that text and tables cannot do. Ideas may even be formulated as strategic narratives which are easier to understand and present to others. Visual, graphic presentations are forceful and may be an important tool to present and discuss complex scenarios and make new ideas and thoughts viable. Five examples of strategic options are discussed here:

#### (I) Stay and consolidate the current position

This is a common option when the current position in the scientific landscape is considered both optimal and desired. In most cases, however, there will be other research groups occupying the same scientific territory. If the groups are very similar and compete for the same funding, maintaining status quo—without strengthening the position—may not be a long-term viable option. Compare, for example, the sections of Biodiversity (ECOS) and Eco-informatics (BIOL) (see Figs 15 and 16). Here strategic thinking is called for to discuss how best to deal with the obvious scientific overlap. If competition is the selected option, a group may decide to recruit new researchers or to strengthen the competences of the existing staff. Where the groups belong to the same university, it might be wiser to collaborate or try to diversify research or main tasks to minimize the undesired effects of internal competition.

#### (II) Expand into new areas

In some cases a research group may have limited research activity in parts of the science map where it want to be present. Either because the unoccupied research area is important to fulfil the traditional main tasks of the group (e.g., teaching or advisory work), or because there are new, interesting research themes that could expand the competences of the group and make it more competitive. This could be accomplished by letting members of the group specialize in the new area, recruiting an established researcher from the research specialty or setting up a formalized collaboration with another research group from that domain.

#### (III) Merge and fuse research specialties

The history of science has many examples of how new scientific specialties emerge when two unrelated research areas interact and amalgamate. Traditional lab-based and experimental zoophysiology, for example, has fused with more field-oriented population ecology and resulted in a research specialty known as ecophysiology. Another example is the recent advances in DNA sampling techniques which have made it possible to extract DNA from environmental samples (eDNA) and obtain precise information about species present in a given area and thus boosting biodiversity studies. In this way, interdisciplinarity contributes to the discovery of new knowledge and, in some cases, become research specialties in their own right.

#### (IV) Explore beyond the border of the reference map

A science map is usually based on a limited sample of papers to keep a relatively narrow scope and balance overview and detail as discussed above. In some cases, therefore, a detailed science map (as the one presented in this study) may conceal interesting scientific territory that could inspire to new creative ideas for science development. Biologists, for example could look to engineering for hardware- or equipment improvement, to data science for new data mining or machine learning techniques, or to social sciences to include the human dimension in research to improve the societal impacts of advisory work.

#### (V) Invent new territory

Innovation and revolution are in the core of science where new territory is discovered and existing truths and theories are overturned. Resurrection biology (or de-extinction) is a recent example from biology where cloning in theory can be used to bring extinct animals back to live and re-introduced into the wild. Another hypothetical example is population ecology of artificial life. It is obvious that such areas cannot be mapped before they exists, but perhaps the mapping of existing specialties could inspire and initiate new ideas that in time could evolve into real research specialties.

Options (I) and (II) do not change the reference map itself, but fortify or expand the positions of the groups that inhabit it. Options (III-V), however, will gradually contribute to a rearrangement and change of the reference map. The position of keywords will shift and the layout of the science map will change when previously separate research subjects merge into something new, or when completely new research specialties arise. This ‘continental drift’ of research specialties and paradigms is an innate part of science development [22].

### Suggestions for further development

This paper uses science maps in an attempt to ‘make sense of scholarly knowledge and expertise in a comprehensive and timely fashion’ as suggested by Börner [62]. Science mapping is not a new discipline, but many previous studies have a global or large-scale perspective on science mapping [17, 22, 62]. This study has a much narrower and local focus and investigates the applicability of science mapping for a research department to guide strategic thinking, support scientific curiosity within and beyond the department and thus promote collaboration, interdisciplinarity, creativity and outreach. There are, however, many other possibilities and potential uses of science maps to explore and a few examples are listed below:

#### Use larger scale reference science maps to gain perspective

The science map developed for this study is based on scholarly papers written or co-written by the current research staff in biology at Aarhus University. It is clearly a restricted sample that doesn’t cover the whole width of the biological science domain. Any map must balance overview and detail and it is very difficult to cover both at the same time. The local map in this study, consequently, cannot show how much of the full scientific realm of biology ECOS and BIOL cover jointly. An obvious solution to this problem is to create general reference maps on a larger scale based on a representative sample of papers from the current global front of biology research. The sampling itself, however, is a problem as there is no clear definition of biology and the science specialties it contains. Strategies for selecting a representative sample could combine use of Web of Science subject categories, papers from a representative group of researchers or institutes, papers published by certain journals, or sampling based on keyword searches [25]. A practical solution could involve the production of a series of maps where scope and overview were gradually enlarged.

#### Exploring the dynamics and evolution of a science specialty

From the beginning of the 1990’s, good data are available for science mapping which makes it possible to study the development of science over time and science maps can visualize how profiles of individual researchers, groups or departments change position and distribution over time. It is also possible to create several reference maps from different periods to see how research specialties are formed, how they develop and perhaps disappear again. The evolution of science is a research specialty in its own right [63, 64]. Here, it is of special interest to determine the so-called research front and be able to predict the direction of research, e.g., in order to be a first mover in a new and promising research area. A good example of a keyword that is in the forefront of a recent emerging research area is *climate change*. Since 2005 the use of this keyword has grown almost exponentially [35]. Today it dominates natural sciences and was also the most commonly used keyword in this study.

#### Compare research institutions within a country, a region, or globally

For strategic planning and thinking, it is not only important to know the strong and weak spots of one’s own department or research group; it is equally important to know how potential collaborators and competitors perform. Research groups often compete for funding from the same sources and science map profiles of other research groups may help determine whether or not to compete or collaborate. An example is shown in Fig 13 where the profiles of ECOS and BIOL provide a clear picture of where in the landscape there are overlaps and where there are distinct research areas.

#### Interactions between science and the public

Keywords phrases – except perhaps very narrow and specific terms - are not just part of the scientific jargon and language, but are also used to describe scientific findings or to discuss and address topics or issues of concern by the media and in the public debate. Science maps can be used to study how science reflects the surrounding society and vice versa. A text analysis of, for example, newspaper, internet or other media articles from a given period of time and location, can be projected on a science reference map to show how well research activities reflect societal problems. Science maps can also be used to explore how well a research area is known in the public. There are a few examples of this in the literature. One study analyses how much social media attention results from microbiology research receive [65] and another focuses on papers that include the phrase ‘*water framework directive*’ to discover to what degree current freshwater research reflects the EU Water Framework Directive [42].

#### Recruitment

As described above, a science map can be used to identify areas where recruitment of new staff is necessary. It may also support recruitment by first identifying the relevant area of interest within the science map and the most important keywords associated with it to write a concise and sharp job description. The next step is to identify important research groups and established researchers within the area and use this network actively to target potential applicants. Finally, it is easy to overlay the profile of each applicant to the science map in order to compare and select the candidate that best fits the job description.

These cases are examples of a wider and more dynamic use of science maps to enhance and stimulate the development of science. Science maps are not black boxes that deliver clear-cut answers to complicated problems, but they constitute an important source of information and deliver new angles to improve and facilitate processes of strategic thinking, organization of teams, and for knowledge exchange between science and society.

## Conclusion

The world of science is growing at an unprecedented speed with more and more scholarly papers produced each year. The scientific landscape is constantly changing as research specialties evolve, merge or become obsolete, which makes it very difficult to maintain an updated view of the research activity in a scientific field. The results presented in this study, show that it is possible to extract keyword-based information from a corpus of papers using a standardized and almost automatic procedure to produce a two-dimensional reference map that presents a scientific landscape in a meaningful and recognizable way.

Science maps provide new and often unexpected ways of looking at diverse and complex research landscapes. This study demonstrates that when a reference map is overlaid papers, researchers or research groups at various levels, new and valuable information on the position, breadth and width of the research becomes visible. These patterns and profiles may be used to compare researchers or groups of researchers to find and describe differences, similarities, gaps and overlaps. These profile maps are a valuable source of information and are useful for different purposes: They provide an instant view of the current state of research and deliver precise and detailed information on the type of research carried out at a research institute. The maps show the distribution of staff and research groups and how they are related. Such information makes it easier to find researchers working on a specific subject, to search for potential collaborators, or to support recruitment processes where researchers with a specific profile are needed. The maps are suitable for top-level research management because they may point to areas in need of support where, e.g., older researchers are expected to retire, or where new, emergent research field are beginning to form and gain momentum. Science mapping supports intended (top-down) as well as emergent (bottom-up) strategy processes. The maps do not provide strict answers, but provide alternative views of the intricate structures of science that supplement traditional bibliometric information. This may help foster strategic thinking to enhance overview, knowledge of the field, creativity and thus further and improve science. Science mapping have matured to a degree where it is ready to be included as standard tools in strategy development.
