# Correction: Science maps for exploration, navigation, and reflection—A graphic approach to strategic thinking

**DOI:** 10.1371/journal.pone.0316560

**Published:** 2024-12-26

**Authors:** Flemming Skov

Fig 11 is uploaded incorrectly. Please see the correct Fig 11 and its caption here.

**Fig 11:**
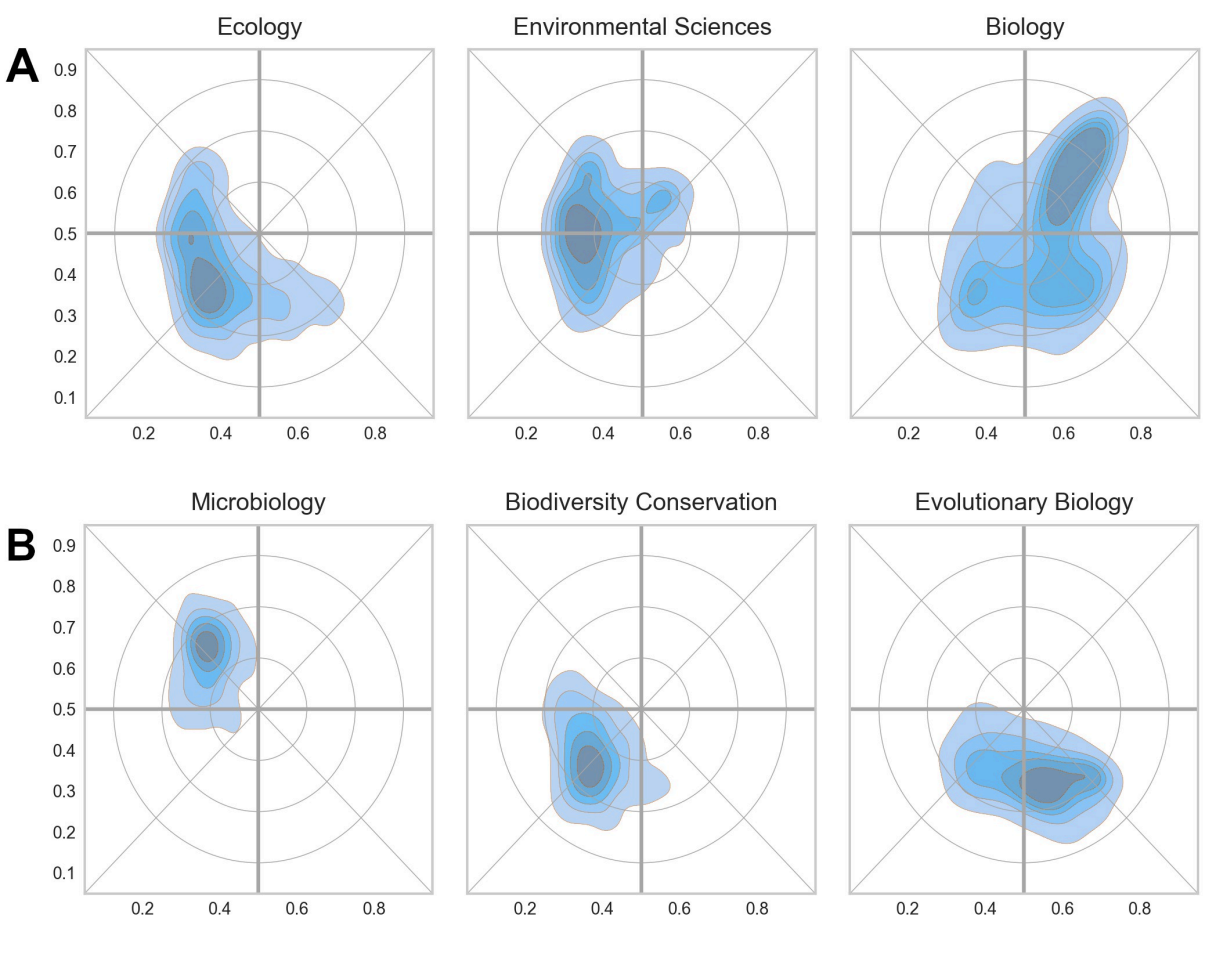
1. Examples of broad (11A) and narrow (11B) Web of Science Subject Categories in the reference landscapes. The color of the shading is not uniform across the figure but is relative to the category in question.

In the Distribution of scientific papers and their Web of Science subject categories subsection of Results, there is an error in the fourth sentence of the fourth paragraph. The correct sentence is: Fig 11 shows the density profiles for Microbiology, Biodiversity Conservation and Evolutionary Biology with centers of distribution in three of the four major quadrants in the map (north-west, south-west and south-east, respectively).
